# SHOOT GROWTH1 Maintains Arabidopsis Epigenomes by Regulating *IBM1*

**DOI:** 10.1371/journal.pone.0084687

**Published:** 2014-01-03

**Authors:** Vincent Coustham, Daniela Vlad, Aurélie Deremetz, Isabelle Gy, Francisco A. Cubillos, Envel Kerdaffrec, Olivier Loudet, Nicolas Bouché

**Affiliations:** 1 INRA, UMR1318, Institut Jean-Pierre Bourgin, RD10, Versailles, France; 2 AgroParisTech, Institut Jean-Pierre Bourgin, RD10, Versailles, France; St. Georges University of London, United Kingdom

## Abstract

Maintaining correct DNA and histone methylation patterns is essential for the development of all eukaryotes. In Arabidopsis, we identified SHOOT GROWTH1 (SG1), a novel protein involved in the control of gene methylation. SG1 contains both a Bromo-Adjacent Homology (BAH) domain found in several chromatin regulators and an RNA-Recognition Motif (RRM). The *sg1* mutations are associated with drastic pleiotropic phenotypes. The mutants degenerate after few generations and are similar to mutants of the histone demethylase INCREASE IN BONSAI METHYLATION1 (IBM1). A methylome analysis of *sg1* mutants revealed a large number of gene bodies hypermethylated in the cytosine CHG context, associated with an increase in di-methylation of lysine 9 on histone H3 tail (H3K9me2), an epigenetic mark normally found in silenced transposons. The *sg1* phenotype is suppressed by mutations in genes encoding the DNA methyltransferase CHROMOMETHYLASE3 (CMT3) or the histone methyltransferase KRYPTONITE (KYP), indicating that SG1 functions antagonistically to CMT3 or KYP. We further show that the *IBM1* transcript is not correctly processed in *sg1*, and that the functional *IBM1* transcript complements *sg1*. Altogether, our results suggest a function for SG1 in the maintenance of genome integrity by regulating IBM1.

## Introduction

Methylation of cytosines and histones are epigenetic modifications found in many eukaryote species, including plants and animals that influence gene expression and transposon mobilization. In contrast to mammals for which DNA methylation occurs predominantly in the CG cytosine context, with the exception of embryonic stem cells [1], plant DNA methylation is found in both symmetrical (CG and CHG where H is A, T or C) and non-symmetrical (CHH) contexts. These three types of cytosine methylations are maintained by different specific pathways in Arabidopsis [2]. The DNA methyltransferases METHYLTRANSFERASE1 (MET1) and CHROMOMETHYLASE3 (CMT3) are required for the maintenance of CG and CHG methylation, respectively [2]. Maintaining CHH methylation depends on both CHROMOMETHYLASE2 (CMT2) [3] and the small RNA-directed DNA methylation (RdDM) pathway, which involves plant specific RNA polymerases (Pol IV and V) and the production of 24-nucleotide small RNAs controlled by the RNA-DEPENDENT RNA POLYMERASE2 (RDR2) together with DICER-LIKE3 (DCL3) [2]. Histones can be methylated in several ways, one of them being at lysine 9 on histone H3 tail (H3K9me), a hallmark of silent chromatin in several organisms, including plants [4]. Methylation at H3K9 can be classified into three different types: mono- di- or tri-methylated, but di-methylation (H3K9me2) is the most common in Arabidopsis. H3K9me2 is catalysed by three histone methyltransferases, KRYPTONITE/SUVH4 (KYP), SUVH5, and SUVH6 [5].

Locus specific CHG and H3K9me2 methylations are intimately connected. Indeed, CMT3 physically interacts with H3K9me2 marks through both its chromo and BAH domains [6] while KYP binds CHG-methylated cytosines through its SRA domain [7]. Altogether CMT3 and KYP participate in a self-reinforcing loop between DNA and histone methylation that is essential for transposons and repeated sequences silencing. Another evidence for an interplay between DNA and histone modifications was the identification of INCREASE IN BONSAI METHYLATION1 (IBM1), a Jumonji C (JmjC) domain protein belonging to the JHDM2 family of H3K9 demethylases that is specific to H3K9 mono- and dimethylation [8]–[10]. *IBM1* was identified in a screen for increased methylation at the *BONSAI* (*BNS*) locus since cytosines of *BNS* in the CHG context are highly methylated in *ibm1*. In both *ibm1*/*cmt3* and *ibm1*/*kyp* double mutants, CHG methylation at *BNS* returns to a WT level, showing that IBM1 counteracts CMT3 and KYP activities at this locus. In fact, epigenome-wide analyses of *ibm1* identified thousands of other genes that are ectopically enriched for both CHG and H3K9 methylations in *ibm1* mutants [9]. Thus, IBM1 prevents genes from being targeted by the machinery that silences repeated elements and transposons. Additionally, IBM1 acts on components of the RdDM pathway itself through direct targeting of *RDR2* and *DCL3* genes [11].

Here, we report the identification of a novel player, named *SG1*, in the regulation of DNA and histone methylations. Initially identified as a major QTL candidate for shoot and root growth in several segregating populations, the *sg1* mutant exhibited pleiotropic developmental defects with a higher penetrance in successive generations similarly to previously reported mutants involved in chromatin modifications. *SG1* encodes a protein with two putative domains, one of them, a Bromo-Adjacent Homology (BAH) domain being found in several chromatin binding proteins of eukaryotes. A genome-wide methylation analysis in the *sg1* mutant background using bisulfite sequencing showed that a large number of gene body sequences were enriched in CHG methylation, similarly to previous reports on *ibm1*. We also found an increase in H3K9me2 levels within the gene bodies of all CHG hypermethylated candidates tested. We confirmed through a genetic approach that the *sg1* mutant phenotype is suppressed by *cmt3* or *kyp* mutations, but not *ibm1*. We analysed the expression of some targets of SG1 and in particular genes involved in the RdDM pathway, but we found no correlation between an increase in gene body CHG methylation and a lack of function. Finally, we show that the accumulation of full-length functional *IBM1* mRNAs is compromised in *sg1*, which can be complemented by the introduction of a transgene producing the long version of *IBM1* transcripts. Altogether, our data reveals a new player in the maintenance of Arabidopsis DNA and histone methylations with an essential role in epigenome stability.

## Results

### Identification and Characterization of *sg1* Mutants

In a previous report we described a quantitative genetic screen to investigate natural genetic variation for shoot growth in *Arabidopsis thaliana* [12]. Among four significant QTLs mapped in the Bur-0 x Col-0 recombinant inbred line population, one QTL named *SHOOT GROWTH1* (*SG1*) was mapped at the top of chromosome 5 (Figure S1 in File S1). Plants carrying a Col-0 allele at *SG1* locus showed drastic shoot growth defects with respect to plants carrying the Bur-0 allele, as confirmed in heterogeneous inbred families (HIF). Fine-mapping analysis using recombinant HIF mapped *SG1* to a locus localized in a 8.5 kb interval comprising a single gene, At5g11470. In parallel, we fine-mapped a QTL detected in the Ct-1 x Col-0 RIL population with a major effect on root growth (named *ROOT GROWTH1*; *RG1*) down to the same region and gene (Figure S2 in File S1).

**Figure 1:**
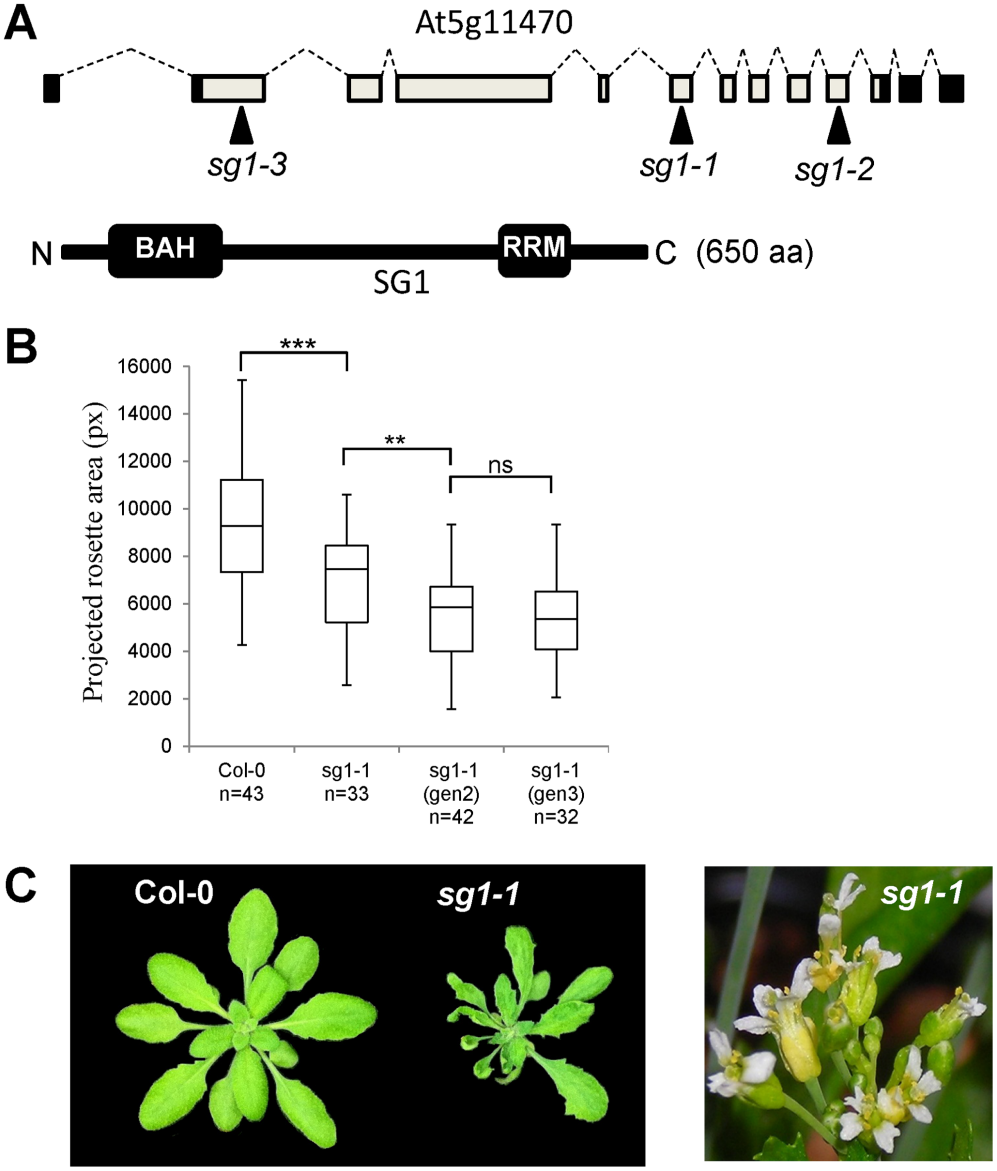
*SG1* encodes a protein with domains involved in chromatin modification. (A) Top: *SG1* gene structure showing UTRs (black boxes), exons (clear boxes), introns (dashed lines). The position of *sg1-1* mutation (cytosine at position 2,954 relative to ATG) and *sg1-2* or *sg1-3* T-DNA insertions are indicated by plain arrows. Bottom: protein structure showing the two putative BAH and RRM domains (black boxes). (B) Analysis of the projected rosette area (pixels) of 15 days-old seedlings grown *in vitro*. *sg1-1*(gen2) : progeny of *sg1-1*; *sg1-1*(gen3) : progeny of *sg1-1*(gen2). n: number of individuals analyzed for each genotype. **p<0.01, ***p<0.001, ns: not significant. (C) Left panel: *sg1-1* phenotype of a 25 days-old plant (right) compared to WT Col-0 plants. Right panel: *sg1-1* floral defects. Note the aberrantly shaped petals, incorrect organs number.

A sequence analysis on *SG1* did not evidenced obvious DNA polymorphisms common to stock accessions Ct-1, Bur-0 and Col-0, however, different gene model predictions for the *SG1* gene were found in the databases (Figure S3 in File S1). Hence, we decided to sequence the *SG1* cDNAs from the RILs themselves to exclude any possible mRNA mis-splicing that could account for the phenotype. Surprisingly, we found a single C to T mutation in the *SG1* cDNA from plants of the mapping populations carrying Col-0 alleles, compared to the sequences of parental accessions. Analysis of the cloned *SG1* cDNA, that matches EuGène prediction [13] with slight variations in regard to TAIRv10 prediction (Figure S3 in File S1), suggested that this *sg1-1* mutation identified in the RILs occurs in the sixth exon of the gene (Figure 1A). By analyzing the original seeds of the Col-0 parent used to produce the RILs, we confirmed the presence of the *sg1-1* mutation compared to the Col-0 reference sequence. We concluded that *sg1-1* is a non-silent spontaneous mutation that appeared in our Col-0 parental line before we developed our RIL sets.

In the original Col-0 accession carrying the *sg1-1* mutation at the homozygous state, we observed that the phenotype was not fully penetrant since we identified both plants with no or subtle phenotype in addition to plants with reduced shoot size and a phenotype that increases throughout generations (Figure 1B and Figure S4 in File S1). The shoot growth reduction phenotype was associated with dark green plants and pleiotropic developmental abnormalities including altered leaf morphology (narrow and serrated), and flowers with more petals, some being fused or with an altered morphology (Figure 1C). After at least four generations at the *sg1*-homozygous state, plants exhibited a terminal phenotype with small deformed leaves associated with strong sterility. We then isolated two T-DNA knockouts (*sg1-2* and *-3*) with insertions localized within the *SG1* coding sequence (Figure 1A). Both mutants were similar to *sg1-1*: the severity of the phenotype varied between individuals and became stronger in successive generations to reach a complete sterility from the 4^th^–5^th^ generation (Figure S5 in File S1).

*SG1* (At5g11470) encodes an unknown protein with two predicted domains: an RNA Recognition Motif (RRM) and a Bromo-Adjacent Homology (BAH) domain (Figure 1A). The *sg1-1* mutation results in a Gln to STOP codon, leading to a potentially truncated protein with no RRM domain. While the Arabidopsis genome encodes a unique SG1 protein, we found a total of 21 proteins that contain a BAH domain (Figure S6 in File S1), including well characterized DNA methyltransferases like MET1 and CMT3, pointing towards a function for SG1 in chromatin regulation.

### Gene Body CHG Methylation and H3K9 Marks are Impaired in *sg1*

**Figure 2:**
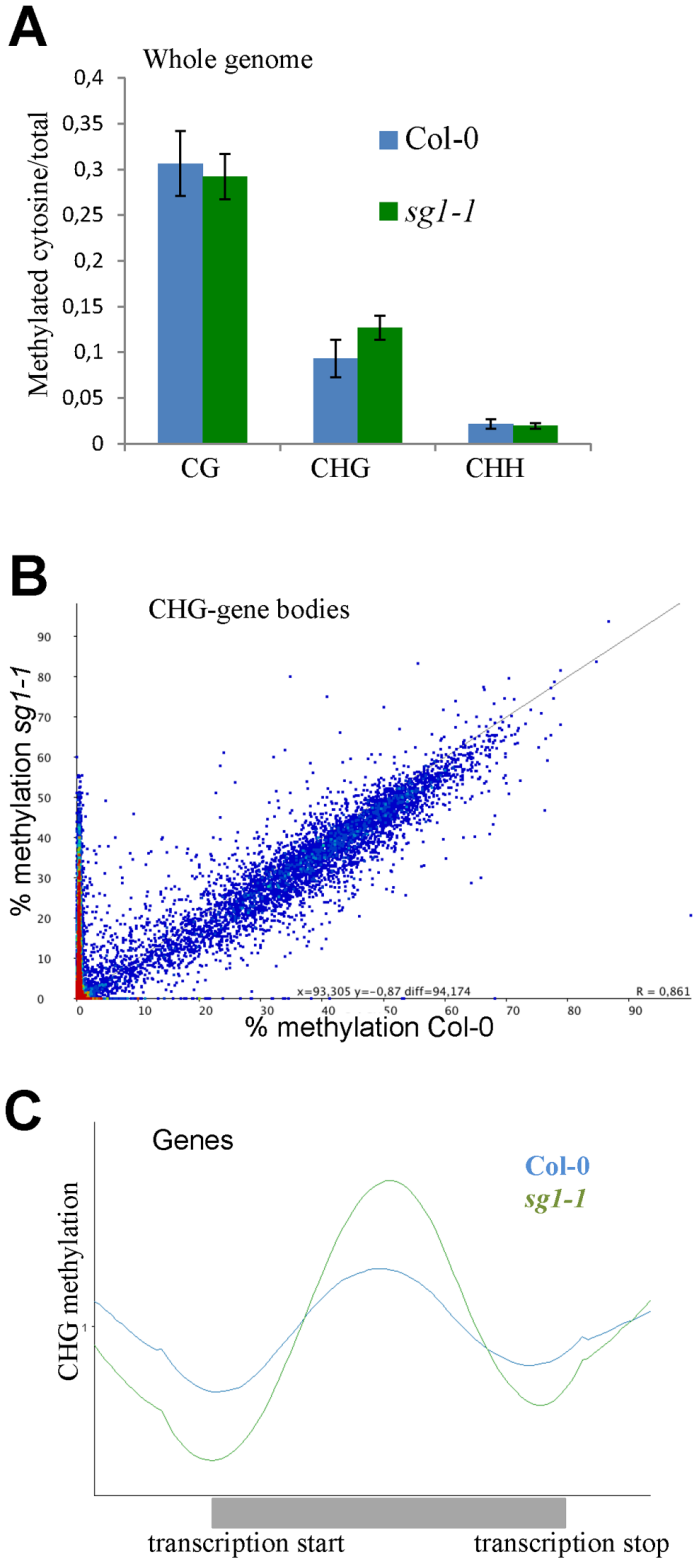
*sg1-1* methylome analysis. (A) Whole genome analysis of cytosine methylated in all three contexts of methylation (CG, CHG and CHH) in Col-0 and *sg1-1* mutants. Error bars indicate the SD between two biological replicates. (B) Scatter plot representing the percentage of CHG methylation calculated for each gene in *sg1-1* and Col-0. Hypermethylated genes in *sg1-1* are visualized by the dense red area. (C) Graphic representation of the average distribution of CHG methylation in gene bodies.

The pleiotropic and generational nature of the phenotype is reminiscent of those observed in mutants disrupted in chromatin modification pathways such as *ibm1* [10], *met1* [14] or *ddm1* [15]. Thus, we analysed the methylome of *sg1-1* mutant through Illumina deep sequencing after bisulfite conversion, in comparison to the Col-0 background. The coverage of sequenced Arabidopsis methylomes was 30X with an error rate of 0.4% (Table S1 in File S2). First, we calculated the percentage of methylation for cytosines in either one of the three contexts CG, CHG or CHH. Genome-wide, we observed no significant differences between Col-0 and *sg1-1* for cytosines methylated in CG or CHH, while *sg1-1* seemed to contain more methylated CHG than Col-0 (Figure 2A; Table S2 in File S2). This was further confirmed when we calculated the percentages of methylation in 200 bp-windows (with a 50 bp overlap) to scan the whole-genome. The *sg1-1* mutant contained a large number of regions enriched in methylated-CHG compared to Col-0, while no such large-scale contrast was observable for cytosines in the CHH or CG contexts (Figure S7 in File S1). To determine whether CHG regions highly represented in *sg1-1* corresponded to genes or other parts of the genome, we calculated the percentage of cytosine methylated per gene and we found that many genes of *sg1-1* were enriched in CHG methylation compared to Col-0 (Figure 2B). Gene body-methylation in the CG context was similar between Col-0 and *sg1-1* (Figure S8A in File S1), while -overall- genes seem to be slightly over-methylated in the CHH context in *sg1-1* (Figure S8B in File S1). The average distribution of CHG methylation over genes indicates that genes are more methylated in their core bodies (Figure 2C). We found no systematic contrast in methylated-cytosine content between *sg1-1* and Col-0 transposable elements (Figure S9 in File S1).

**Figure 3:**
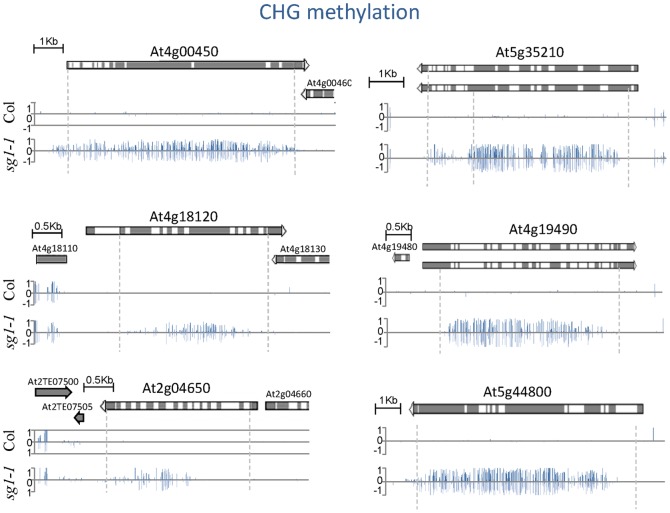
Gene-body CHG hypermethylation in *sg1-1*. Methylation on top (positive values) and bottom strand (negative values) across the coding sequence of the genes indicated is shown. Data are based on methylome results. The corresponding gene models are shown according to TAIR v10.

**Figure 4:**
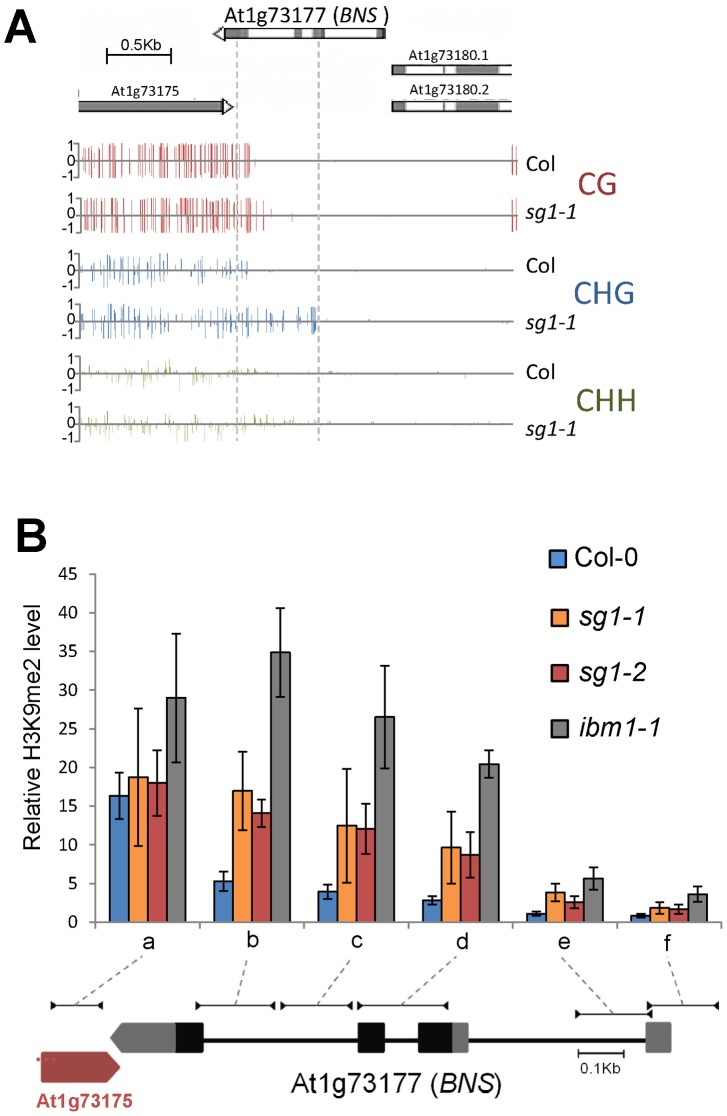
Methylation analysis at *BONSAI* in *sg1-1.* (A) Methylation in the three cytosine contexts near *BNS*. The spreading of DNA methylation from the adjacent LINE element (At1g73175) is visible in *sg1-1*. (B) Histone H3K9me2 accumulation in *BNS* determined by ChIP analyses. The correspondence of genomic regions tested by ChIP is shown on a gene schematic based on TAIR v10. Error bars are SEM from at least three biological replicates.

We fixed a minimal methylation ratio of two from Col-0 to *sg1-1* to classify genes as hyper- (2x increase in *sg1-1*) or hypo-methylated (2x decrease in *sg1-1*; see Materials and Methods). Under these criteria, we found less than 100 genes that were hypomethylated in either one of the three contexts, one gene hypermethylated in the CG context, 0 in CHH and over 3,300 in the CHG context (Table S3 in File S2). Genes found to be CHG-hypermethylated were equally distributed among the five Arabidopsis chromosomes (Figure S10 in File S1). We selected six genes from this list to analyze in more details the distribution of the methylated cytosines within gene bodies. We confirmed that all these genes were significantly enriched in CHG-methylated cytosines in *sg1-1* mutants compared to Col-0 throughout the gene sequence (Figure 3), while no differences were observable for CG methylated cytosines, and a slight increase for some of them in the CHH contexts (Figure S11 in File S1). Altogether, our results indicate that SG1, very similarly to IBM1, protects certain protein-coding genes from ectopic CHG methylation. Notably, as observed in *ibm1-1* mutants [10], CHG and -to some extent- CHH methylation, extend from an adjacent transposon into the *BONSAI* locus in *sg1-1* (Figure 4A).

**Figure 5:**
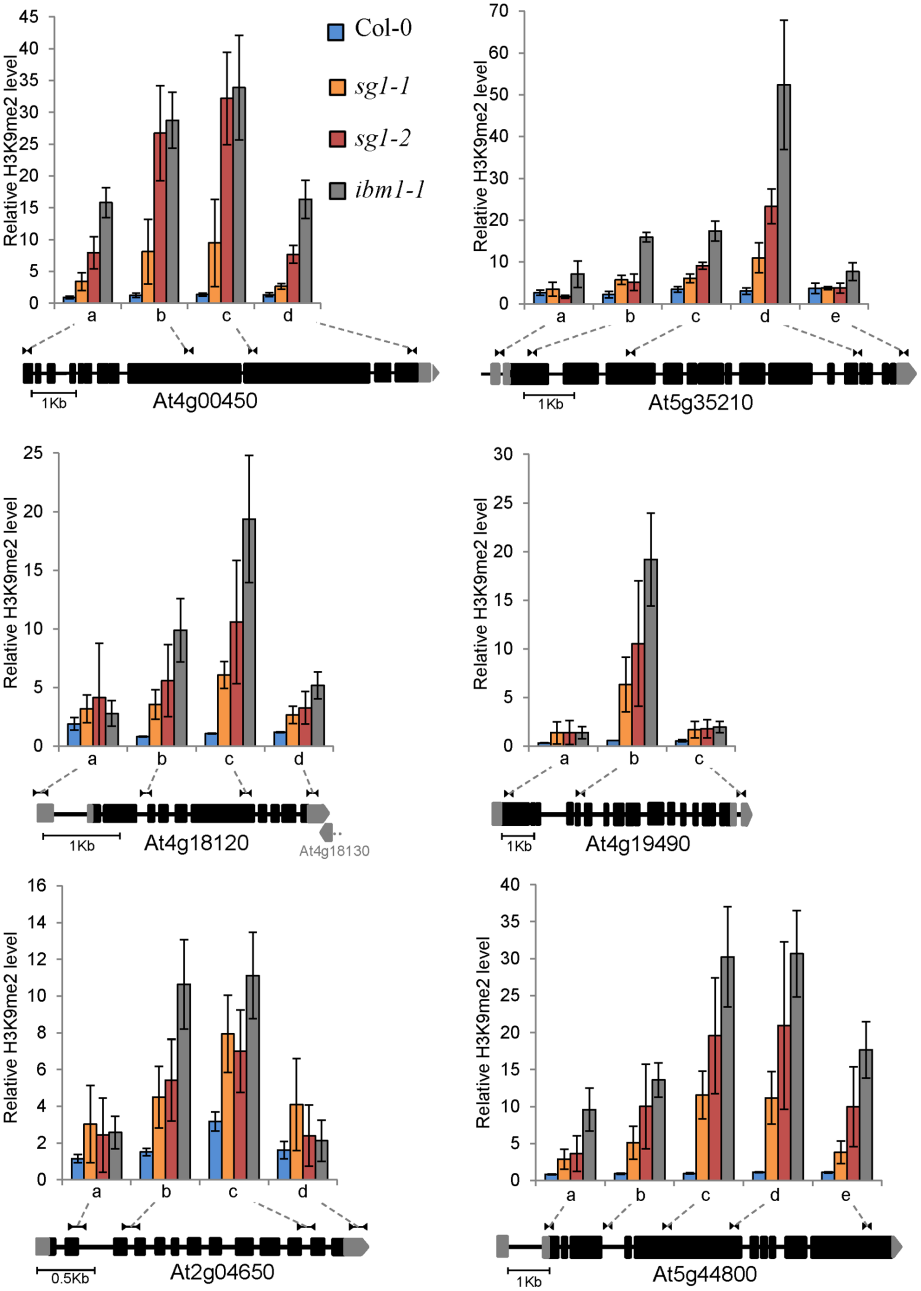
Gene-body H3K9me2 accumulation in *sg1-1* and *ibm1* mutants. H3K9me2 profile across six genes mentioned in Figure 3, in WT Col-0, *sg1-1*, *sg1-2* and *ibm1-1* plants. The correspondence of genomic regions tested by ChIP is shown on a gene schematic based on TAIR v10. Error bars are SEM from at least three biological replicates.

Since *IBM1* encodes a histone demethylase catalysing the removal of H3K9 marks, a process tightly linked to CHG methylation, we determined H3K9me2 contents by ChIP analyses in the subset of genes that present elevated levels of CHG methylation (Figure 5). Using several couples of oligos along gene bodies, we observed in both *sg1-1* and *sg1-2* mutant alleles as well as in *ibm1-1*, a significant enrichment of H3K9me2 localized within the center of genes, and to a lesser extent in UTRs (Figure 5). Similarly, the *BNS* gene was enriched in H3K9me2, particularly in regions adjacent to the transposon nearby (Figure 4B). Altogether, our results suggest a function for SG1 in the protection of genes from H3K9me2 and CHG DNA methylation, similarly to that described for IBM1.

**Figure 6:**
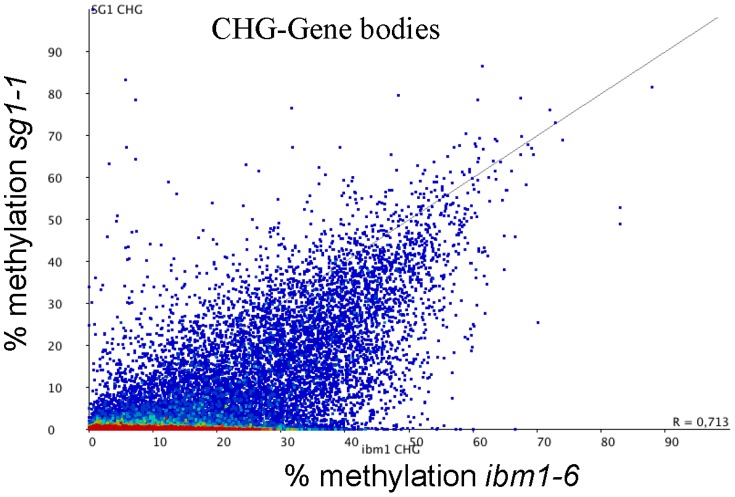
Comparison of *sg1-1* and *ibm1-6* CHG methylated genes. Scatter plot representing the percentage of CHG methylation calculated for each gene in *sg1-1* and *ibm1-6*, according to the data publicly available [16]. Genes solely methylated in *ibm1* are visualised by the dense red area.

Recently, Jacobsen and collaborators revealed the DNA methylome landscapes of several Arabidopsis mutants involved in gene silencing and chromatin remodeling, including components of the methylation pathways [16]. We retrieved the *ibm1* and the corresponding WT methylome sequences to identify the genes differently methylated in *sg1-1* versus *ibm1-6* (SALK_006042). Interestingly, we found that the vast majority of gene bodies differentially CHG methylated in *sg1-1* were also targeted by IBM1, but we also found that many genes more CHG methylated in *ibm1* were unchanged in *sg1-1* (Figure 6). We verified that the CHG methylomes of the WT samples were similar between the two experiments (Figure S12 in File S1).

### CMT3 and KYP act Antagonistically to SG1

**Figure 7:**
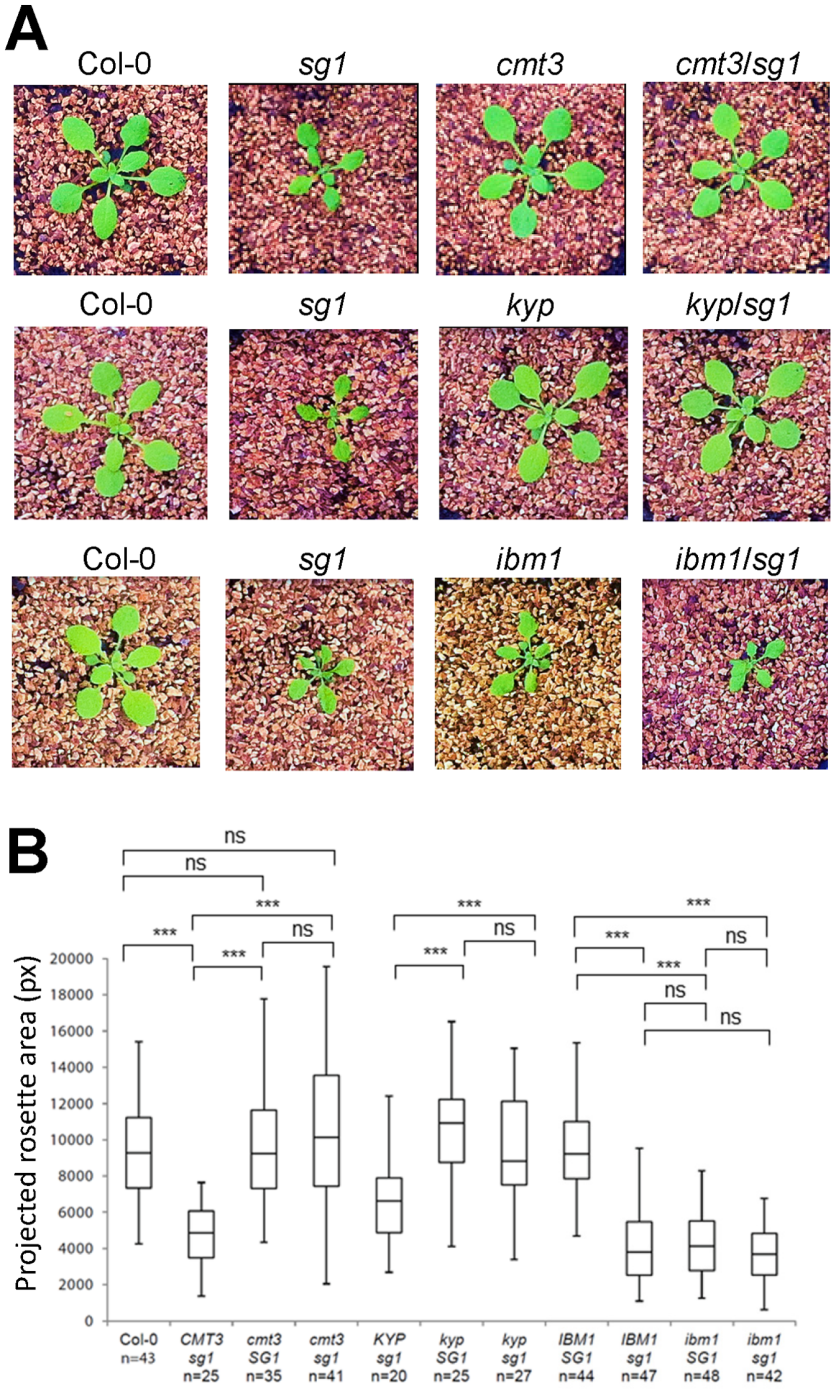
*sg1* phenotype is suppressed by *cmt3* or *kyp* mutations, but not *ibm1.* (A) Pictures of typical 15 days-old seedling corresponding to homozygous double mutants (F4) resulting from the cross between *sg1-2* and *cmt3-11*, *kyp* (line SALK_069326) or *ibm1-1* mutants. Contrast was enhanced for picture visibility. (B) Analysis of the projected rosette area (pixels) of 15 days-old double mutant seedlings (as described in A) grown *in vitro*. n: number of individuals analyzed for each genotype. ***p<0.001, ns: not significant.

IBM1 and KYP have opposite effects on H3K9 marks, and this has a direct impact on CMT3 activity and CHG methylation. Thus, IBM1 restricts CMT3 and KYP activities to transposons and repeated sequences, protecting genes from elevated levels of CHG methylation and H3K9me2. To investigate the relationships between *SG1*, *IBM1*, *KYP* and *CMT3* we crossed *sg1-2* with mutants corresponding to these genes. Both *sg1/cmt3* and *sg1/kyp* double mutants showed a WT phenotype, indicating that either *cmt3* or *kyp* mutations alone can suppress the severe phenotype of *sg1* (Figure 7) and *ibm1* [8]. On the other hand, the double *sg1/ibm1* mutant is phenotypically similar to the *sg1* or *ibm1* mutants (Figure 7). This suggests a function for SG1 in the protection of genes from H3K9me2 and CHG DNA methylations, together with IBM1, and antagonistically to that of CMT3 and KYP.

CMT3 is the methyltransferase that maintains CHG methylation and MET1 is involved in the maintenance of CG methylation. To understand whether perturbing one of the other types of methylation in *sg1* would have some consequences, we combined the *met1* mutation with *sg1*. In the F2 population, we obtained only three plants fixed for both mutations, on a total of 96 plants (Figure S13A in File S1). The F3 progenies of one of these *met1*/*sg1* double mutants had a strong phenotype (Figure S13B in File S1) and were all sterile. Therefore, suppressing the CG-methylation in both genes and transposons (i.e. *met1*), while adding silencing marks in the core of genes (i.e. *sg1*) has a deleterious immediate effect on plant development.

Previous genetic analysis showed that some components of the RdDM pathway, including RDR2, NRPD1A and AGO4, are dispensable for *ibm1*-induced DNA hypermethylation defects, suggesting that de novo DNA methylation of the *IBM1* targets is subject to siRNA-independent regulation [9]. Therefore, we investigated if hypermethylation in *sg1* was also siRNA-independent, similarly to what was reported for *ibm1*. When we combined *sg1* with a mutant lacking the AGO4 function that is crucial for RdDM processes [2], we found that the double *sg1-2*/*ago4-2* mutant was phenotypically very similar to *sg1-2*, suggesting that *sg1*-induced hypermethylation does not depend on components of RdDM (Figure S14 in File S1).

### Levels of mRNAs in *sg1*, Methylation and Consequences on Gene Function

**Figure 8:**
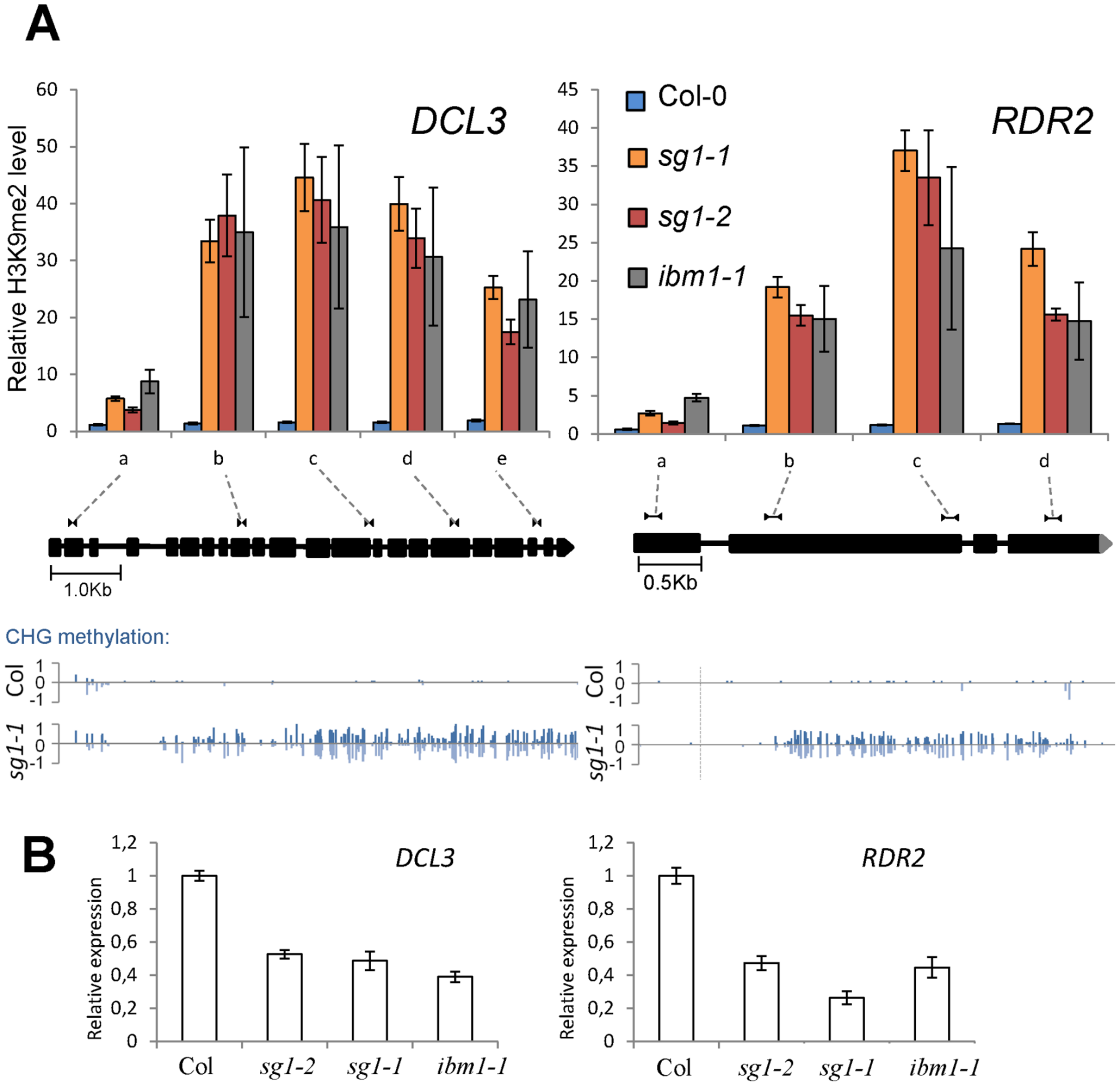
*sg1* and key players of the RdDM pathway. (A) H3K9me2 profile across *RDR2* and *DCL3* genes in WT Col-0, *sg1-1*, *sg1-2* and *ibm1-1* plants. The correspondence of genomic regions tested by ChIP is shown on a gene schematic based on TAIR v10; CHG methylation is presented below. Error bars are SEM from at least three biological replicates. (B) Transcript accumulation of *RDR2* and *DCL3* in *sg1* and *ibm1* determined by qPCR analyses. Error bars are SEM from three replicates.

One of the most intriguing questions regarding the methylation marks that appear in gene bodies of *sg1* or *ibm1* mutants is whether they have direct consequences on gene expression, and ultimately on the function of these affected genes. Recently, IBM1 was suggested to act indirectly on the RdDM pathway by targeting both *RDR2* and *DCL3*, reducing their expression, correlating with a final up-regulation of some RdDM-targets [11]. We analyzed the H3K9me2 enrichment at *RDR2* and *DCL3* locus, by ChIP analysis. We found that, like *ibm1*, *sg1* mutants contain higher levels of H3K9me2 and cytosines methylated in the CHG context at both locus compared to the Col-0 WT plants (Figure 8A); this correlated with more subtle modifications of CHH cytosine methylation but not with CG methylation (Figure S15 in File S1). We confirmed that both *RDR2* and *DCL3* gene expression is reduced by half in *ibm1* and we found similar results for the two *sg1* alleles tested (Figure 8B). We also found other members of the RdDM pathway with a CHG-methylation status affected in *sg1*, including *NRPD1A*, one of the polIV subunits (Table S4 in File S2). However, even if some of the RdDM key players are impacted by *sg1*-induced methylation defects (at CHG, CHH and H3K9 levels), the functional consequences on the RdDM pathway activity remain to be determined.

We tested the expression of several other targets having gene bodies enriched in both H3K9me2 and CHG, and found genes (At4g00450 and At5g35210, see Figures 3 and 5) whose expression was not modified between *sg1-1* and WT plants (Figure S16 in File S1). We concluded that *sg1-*induced methylation defects are not necessarily correlated with changes in the final quantity of transcripts detected, or that *sg1* may induce other changes such as transcript processing alteration that were not detected in our gene expression assays.

Since *sg1* and *ibm1* phenotypes are very similar, both molecularly and morphologically, we thought that the function of IBM1 could be compromised in *sg1* mutants. The *IBM1* gene encodes two different transcripts and only the longest one (*IBM1-L*) is functional and can complement an *ibm1* mutant [17]. We first tested whether the *sg1* phenotype could be the result of *IBM1* transcript misregulation. To this end we examined by qRT-PCR the proportion of the *IBM1-L* mRNA product relative to total *IBM1* mRNA product (as defined by the amount of transcript upstream of the large intron heterochromatic region). Interestingly, we found a stronger reduction in the *sg1-1*/WT mRNA ratio corresponding to the *IBM1-L* product relative to the total amount (Figure S17 in File S1). This suggested that a significant fraction of the mRNA produced at the *IBM1* locus in a *sg1* mutant may correspond to the short, non-functional *IBM1-S* product. We then transformed *sg1* mutants with a transgene carrying a full-length functional *IBM1-L* cDNA construct [17]. Both *ibm1-1* and *sg1-1* were complemented by *IBM1-L*, indicating that the phenotype of *sg1* mutants most likely results from the compromised function of IBM1 in *sg1* (Figure S18 in File S1).

## Discussion

### SG1 and IBM1 act in the Same Pathway

In an effort to map molecular determinants quantitatively controlling growth, we identified a new gene, *SG1* (At5g11470), coding a protein with domains found in chromatin regulators. We identified the *sg1-1* allele corresponding to a recent spontaneous point mutation in the *SG1* coding sequence from the specific Col-0 parent line used to build our mapping population. We also isolated two additional T-DNA alleles (*sg1-2* and *-3*) allowing us to determine that *sg1*-related phenotypes became apparent from the second homozygous generation. All *sg1* mutants present similar phenotypes appearing stochastically, namely growth defects, abnormally shaped organs (including flowers and leaves) and ultimately leading to sterility. The penetrance and severity of the phenotype increase across generations, similar to phenotypic defects previously reported for *ibm1* [10]. Since IBM1 is controlling H3K9 methylation, with consequences on the levels of cytosines methylated in the CHG context, we performed a high-throughput methylome sequencing of *sg1-1* mutants, showing that gene bodies are CHG-hypermethylated in the mutant background (Figure 2B and C) while transposable elements are not affected (Figure S9 in File S1). This was correlated with an enrichment of the heterochromatin mark H3K9me2 at gene bodies of all CHG hypermethylated candidates tested in our study (Figures 3, 4, 5 and 8A). These results strongly suggest that SG1 is working closely to IBM1, indeed, similar enrichments within gene-bodies have been reported in an *ibm1* mutant context [9]. Altogether, these observations suggested a function for *SG1* in the maintenance of genome integrity, likely at the chromatin regulation level, together with IBM1. Recently, two other *sg1* alleles were identified, namely *ibm2* and *asi1* [18]–[19]. In agreement with our hypothesis for SG1 function, the authors showed that SG1/IBM2/ASI1 is required for the processing of long transcripts over intronic heterochromatic marks. Given the presence of an intragenic heterochromatin region in the large intron of *IBM1*, this conclusion is supported by findings that *IBM1* full-length transcript was decreased in the *sg1* mutant compared to the short transcript (Figure S17 in File S1) and that a functional *IBM1* transcript complement *sg1* (Figure S18 in File S1). SG1/IBM2/ASI1 might indeed function by promoting the use of distal polyadenylation sites over proximal ones located in large introns [18]–[19].

SG1 contains two predicted domains, one of them, the BAH domain, being found in various chromatin regulating factors. In plants, one of the two BAH domains of MET1 for instance can physically interact with the C-terminal region of the histone deacetylase HDA6, [20]. Interestingly, BAH domains can also directly recognize histone marks. A component of the Origin of Replication Complex-1 (ORC1) can bind in a specific way H4K20me2 marks that are enriched at replication origins in mammalians [21]. In maize, the homolog of CMT3 binds H3K9me2 marks via a dual recognition through both the BAH domain and the chromodomain of the protein [6]. Therefore SG1 may be recruited to the chromatin via its BAH domain. In addition, SG1 contains a RRM motif shared by a large number of RNA-binding proteins that could also mediate the targeting of SG1 to particular loci. Accordingly, Wang *et al*. showed *in vitro* that SG1/IBM2/ASI1 has RNA-binding properties, and that SG1/IBM2/ASI1 is enriched at intronic heterochromatin regions [19]. The authors proposed a model in which SG1/IBM2/ASI1 is binding to intronic heterochromatin regions and nascent pre-mRNA via its BAH domain and RRM motif respectively, but whether SG1/IBM2/ASI1 BAH domain is directly involved in heterochromatin recruitment remains to be determined.

By comparing our methylome data with the one published recently for *ibm1* mutants [16], we observed that most IBM1 target genes are also affected in *sg1* mutants, while IBM1 has additional targets (Figure 6). However, this does not necessarily mean that IBM1 has a broader range of target genes compared to SG1. This observation might be the consequence of a generational effect since the phenotypes of *sg1* and *ibm1* mutants become more deleterious after several generations. We suspect that the *ibm1* mutant used in the recent study of Jacobsen and coworkers [16] had been accumulating gene methylation for more generations than the *sg1* mutant used in our methylome analysis. In regions where both *ibm1* and *sg1* mutations affect the same genes, we observed that the CHG methylation in *sg1* seems to be more localized to the center of gene bodies. Contrariwise, the CHG methylation in the *ibm1* mutant, while being also localized to gene bodies, seems to be more extended toward the UTRs (Figure S19 in File S1). It is tempting to speculate that generations after generations, the methylation that first invade specifically certain regions of the genes slowly spread to adjacent regions in plants lacking IBM1 or SG1 function. We also detected genes that are methylated in *ibm1* while being almost free of CHG methylation in the *sg1* background (Figure S20 in File S1). Since the phenotype is strongly correlated with the number of generations, it is possible that secondary targets, not impacted in the first generations, are gradually becoming methylated in the progenies, increasing the deleterious effects of the *ibm1* mutation. We currently do not know how methylation acts on mRNA accumulation, but the lethal effects observed after several generations indicate that the constant and possible increasing invasion of the methylation have strong consequences on plant development. To further confirm these hypotheses, methylome analyses of both *sg1* and *ibm1* mutants will have to be compared for the same generations.

### Interconnection between Different Methylation Contexts of Gene Bodies

We have shown that the maintenance DNA methyltransferase CMT3 or the H3K9 methyltransferase KYP are epistatic to *SG1*. Both *cmt3* or *kyp* mutations suppress the phenotype of *sg1*, similarly to what was reported for *ibm1* and suggesting that SG1 functions antagonistically to CMT3 or KYP. In addition to KYP, two other methyltransferases are found in Arabidopsis (SUVH5 and 6), but since the suppression of KYP can restore the WT phenotype in a *sg1* background, it is likely that ectopic H3K9me2 marks deposition in gene bodies of *sg1* mostly involves KYP. The double *ibm1/sg1* mutant displayed similar phenotypes to a single *sg1* or *ibm1* mutant, confirming that IBM1 and SG1 act in the same pathway. Altogether, the interplay between the self-reinforcing CMT3/KYP loop and SG1/IBM1 maintains the Arabidopsis epigenome integrity to ensure correct expression of genes. How SG1 functions to prevent CMT3 and KYP activities at a given locus remains unclear, as well as the initial starting signal that first promotes their activities in genes. But the self-reinforcing loop and the subsequent spreading of the methylation generations after generations seem to be key elements to explain the increase invasion of genes by silencing marks. Recently, DDM1, encoding a chromatin remodeling factor, was shown to restrict CMT3/KYP activities at *BNS* and many other locus. The methylation at *BNS* in all cytosine contexts in a *ddm1* background depends on KYP and CMT3 since the corresponding mutants individually suppress this methylation [22]. In *ddm1*, residual methylation from the adjacent LINE element spreads to *BNS* after several generations, and through the repeated action of both CMT3 and KYP, exactly as in *sg1* or *ibm1* mutants.

The activity of IBM1 seems to be more prone to target actively transcribed genes [9] and the functions of IBM1 and SG1 are essential to keep gene bodies free of CHG methylation. Paradoxically, these genes are not exempt of methylation in the WT, but in a CG-context (Figures S11 and S15 in File S1). The presence of CG-methylation in actively transcribed genes has been described for years, with a role still undetermined at present. Several hypotheses are being examined to discover possible functions for that methylation, some converging towards a potential role in regulating splicing [23]. SG1 could possibly target CG-methylated genes in WT plants, and such targeting could rely on active transcription, or CG-methylation of gene bodies, or both, by direct binding of SG1 to proteins involved in these processes. Intriguingly, even if methylation invades some genes in *sg1* or *ibm1*, it does not prevent their transcription, or at least it does not change the level of transcripts detected by qPCR which is only tested through small transcript sections of 200 bp. The links between transcription or splicing and methylation (CG, CHG or H3K9me2) remains to be investigated further but *sg1* mutants could be appropriate candidates to clarify that point in plants.

Another intriguing observation is that certain CHG-enriched gene bodies of *sg1* seem to be also slightly enriched in CHH methylation, compared to the WT (Figures S11 and S14 in File S1); an observation also true for *ibm1* mutant plants. Part of the CHH methylation depends on the RdDM pathway and the production of sRNAs, but the ectopic CHH methylation in *sg1* is unlikely due to a stimulation of the RdDM pathway and a production of sRNAs from genic regions: first, *ago4/sg1* double mutants are phenotypically similar to *sg1*, indicating that disrupting the RdDM pathway has limited consequences on *sg1* plants and, second, it is unlikely that *sg1* mutants and WT plants have genome-wide dissimilar contents of sRNAs, since *ibm1* and WT plants have not [11]. However, recent evidences point toward the crucial role of CMT2 in maintaining the CHH methylation that is independent on RdDM [3]. The authors hypothesized that CMT2 might bind H3K9me2 marks, exactly like CMT3, to target DNA and methylate cytosines in the CHH context. Genes enriched in H3K9me2 methylation are then also probably targeted by CMT2 in *sg1* and *ibm1*, explaining their enrichment in CHH. H3K9me2 marks could therefore be linked to two contexts of methylated cytosines, CHH and CHG. Further work is required to shed the light on the mechanisms of interplay between SG1 and IBM1, as well as to understand the functional consequences of genic non-CG methylation.

## Materials and Methods

### Plant Material and Growth Conditions

*A. thaliana* RILs and HIFs were obtained from the INRA Versailles collection (http://publiclines.versailles.inra.fr/) and described before [24]–[25]. *sg1* T-DNA mutants were obtained from the Arabidopsis stock center: *sg1-2* is SAIL_310B06 and *sg1-3* corresponds to GK_045A07. In addition, the following Arabidopsis mutants were used: *ago4-2* [26], *cmt3-11* [27], *kyp* (SALK_069326) [28], *met1-1* [14], and *ibm1-1* [10]. Projected area analyses of leaves were performed as previously described [12].

### Whole-genome Bisulfite Sequencing and Analyses

Col-0 and *sg1-1* homozygous seeds were grown in long day conditions for 25 days in culture rooms and 5 to 25 µg of genomic DNA were extracted using a CTAB phenol-chloroform extraction protocol followed by RNAse treatment (Qiagen). Bisulfite treatment, library preparation and whole genome sequencing were performed at BGI (Shenzhen, China) using Illumina’s recommendations and HiSeq technology for paired-end 91 bp reads. We sequenced the methylome of two biological replicates for the WT Col-0 and two for *sg1-1*. The sequencing results are described in Table S1 in File S2.

For sequencing analysis low-quality reads (q <30) were discarded and clean reads were mapped to the Col-0 *Arabidopsis thaliana* TAIR 10 reference genome using the Bowtie and Bismark softwares with options ‘-q -n 3–non directional’. Subsequent analyses were done with SeqMonk (Babraham Bioinformatics - SeqMonk Mapped Sequence Analysis Tool by Simon Andrews). Only cytosines covered by at least 5 unique reads were retained. For genome-wide analyses, we defined windows of 200 bp overlapping by 50 bp intervals to calculate the percentages of methylation along the genome. To determine differentially methylated genes, a Fisher’s exact test was performed comparing methylated and unmethylated counts. We then corrected nominal *P*-values and estimated the corresponding *q*-values to retain genes with *q*-values <0.05. For *ibm1-6* and the corresponding WT Col-0, we retrieved the raw data from NCBI (GEO query: GSE39901) to align it as described for the data obtained in the present study.

### Chromatin Immunoprecipitation (ChIP) and Real-time Quantitative PCR (qPCR) Analyses

ChIP assays were performed as previously described [29] on the aerial parts of 25 days-old seedlings using anti-histone H3 dimethyl lysine 9 antibody from Abcam (Catalog No. ab1220) and anti-histone H3 antibody from Abcam (Catalog No. ab1791). All ChIP experiments were quantified by qPCR using primers listed in Table S5 in File S2. *SHOOT MERISTEMLESS* (*STM*) was used as the internal control for the ChIP experiments. Data are represented as the ratio of (H3K9me2 gene*/*H3 gene)/(H3K9me2 *STM*/H3 *STM*). Experiments were repeated in at least three biological replicates.

### Gene Expression Analysis

Total RNA was isolated from the aerial parts of 15 days-old seedlings using the RNeasy Plant Mini kit (Qiagen) followed by a DNAse treatment (Fermentas). RT-PCR was performed on 500 ng of total RNAs with the M-MLV reverse transcriptase (Fermentas) and cDNAs were diluted 10 times. 5 µl were used for qRT-PCRs using a CFX96 real-time PCR machine (BioRad) with a SYBR solution (Eurogentec) using the primers listed in Table S5 in File S2. Expression levels were normalized against the Arabidopsis *UBC21* gene (At5g25760). Experiments were repeated in three biological replicates.

## Supporting Information

File S1 **Contains: Figure S1:** ***SG1*** **fine mapping.** (A) QTL mapping for shoot size in the Bur-0 x Col-0 RIL set identifies a major QTL on chromosome 5 [12]. (B) Fine-mapping of the major-effect locus to 8 kb around At5g11470 on chromosome 5. **Figure S2:** ***RG1*** **fine mapping in the Ct-1 x Col-0 maps to SG1.** QTL mapping for root growth (total root length) in the Ct-1 x Col-0 RIL set (A) reveals RG1, a QTL confirmed *in vitro* 10 days after germination (days after germination) (B) and in the greenhouse 20 days after germination (C). Fine-mapping and sequencing revealed that *sg1-1* mutation is responsible for this QTL. **Figure S3:** ***SG1*** **gene models.** *SG1* gene models according to TAIR v10, EuGène and our cDNA sequencing data is shown with UTRs (black boxes), exons (clear boxes) and introns (dashed lines). Left, 5′ end; right, 3′ end. **Figure S4:** ***sg1*** **phenotype at the flowering stage.** Picture of a tray of 40 days-old WT Col-0 plants (left) and *sg1-1* plants (middle, right) derived from a parent exhibiting (a) or not (b) a phenotype. The flowering delay can be seen for *sg1-1* (a). **Figure S5:** ***sg1-2*** **and** ***sg1-3*** **mutants.** (A) Phenotype of plants homozygous for *sg1-2* T-DNA (bottom) and WT Col-0 (top) in two successive generations after fixation at the homozygous state. (B) Analysis of the projected rosette area (pixels) of 15 days-old seedlings grown *in vitro*. *sg1-2*(gen2): second homozygous generation. Each following generation (gen3, gen4 and gen5) descends from the previous one. ***p<0.001, ns: not significant. (C) Picture of a mature *sg1-2* plant. The arrow points one sterile bud that failed to develop into a silique. The frame shows an enlargement of a rosette leaf showing the over-serrated shape. (D) Segregating plants (left) originating from a unique *sg1-3* homozygous parent illustrate the stochasticity of the *sg1* phenotype compared to segregating plants from a unique WT Col-0 parent (right). **Figure S6: Proteins containing Bromo adjacent homology (BAH) domain (IPR001025) in Arabidopsis.** The different domains (according to the InterPro database) are indicated: Agenet (IPR008395), ATPase AAA core (IPR003959), Znf PHD-finger (IPR013083), Znf PHD = finger (IPR019787), Chromodomain (IPR023780), TFIIS centre (IPR003618), TFIIS/elonginA/CRSP70 N (IPR017923), C5 DNA meth (IPR022702) and RRM RNP (IPR012677). **Figure S7: Comparison of the percentages of methylation in 200 bp-windows throughout the genome of** ***sg1-1*** **and Col-0.** Each 200 bp-window is represented by a dot. Windows were designed to overlap by 50 bp and only cytosine position covered by at least 5 reads were considered in the calculation. Reads that were not matching at unique locations were discarded. The percentage of methylation in CG (A) and CHH (C) contexts are similar between Col-0 and *sg1-1*, while CHG methylated regions are over-represented in *sg1-1* (B). **Figure S8: Scatter plots comparing the percentages of methylation in gene bodies of** ***sg1-1*** **and Col-0.** Each gene according to the TAIR10 annotations is represented by a dot. Only cytosine position covered by at least 5 reads were considered in the calculation. Reads that were not mapping to unique locations were discarded. The percentage of methylation in CG (A) are similar between Col-0 and *sg1*, while CHH-methylated genes seem to be slightly more methylated in *sg1-1* (B). **Figure S9: Comparison of the percentages of methylation in transposable elements (TEs) in** ***sg1-1*** **and Col-0.** Each transposable element according to the TAIR10 annotations is represented by a plot. Only cytosine position covered by at least 5 reads were considered in the calculation. Reads that were not matching at unique locations were discarded. The percentage of methylation in CG (A), CHG (B) and CHH (C) are similar between Col-0 and *sg1-1*. **Figure S10: Genomic distribution of the CHG differentially-methylated genes identified in** ***sg1-1*** **.** Only genes that present a 2x increase in CHG methylation are represented (see Mat&Met). One hypermethylated gene corresponds to a black line drawn on one of the five chromosomes of Arabidopsis. Gene-poor centromeres are indicated by a ‘c’. The Chromosome Map Tool of TAIR (www.arabidopsis.org) was used to map the different genes on chromosomes. **Figure S11: Gene-body methylation in** ***sg1-1*** **.** Data is based on methylome results. CG, CHG and CHH methylations are shown on top (positive values) and bottom strand (negative values) across the genes indicated. The corresponding gene models are shown according to TAIR v10. **Figure S12: Comparison of data samples for** ***sg1-1*** **and** ***ibm1-6*** **methylome analyses.** (A) Distribution of the percentages of CHG methylation of genes in different samples: *sg1-1*, *ibm1-6*, the WT Col-0 used in this study and one of the three Col-0 samples (sample #3) used by Jacobsen and colleagues [16]. (B) Scatter plots comparing the percentages of methylation in gene bodies of the Col-0 WT used in this study and one of the three Col-0 samples (sample #3) used by Jacobsen and colleagues [16]. Each gene according to the TAIR10 annotations is represented by a dot. The percentage of methylation in CHG are similar between the two samples. **Figure S13: Phenotype of the double** ***met1-1/sg1-2*** **mutant.** (A) Genotyped F2 plants from the *met1-1*/*sg1-2* cross. We found only three plants fixed for the two mutations (circled in red). (B) F3 plants (descending from a F2-genotyped *met1*/*sg1* plant shown in A) were grown in the greenhouse and pictured after three weeks. **Figure S14: Phenotype of the double** ***ago4-2*** **/** ***sg1-2*** **mutant.** (A) Phenotype of the *sg1-2/ago4-2* mutant grown in greenhouse. (B) Analysis of the projected rosette area (pixels) of 15 days-old double mutant seedlings grown *in vitro*. n: number of individuals analyzed for each genotype. ***p<0.001, ns: not significant. **Figure S15: Gene-body methylation of** ***RDR2*** **and** ***DCL3*** **in** ***sg1-1*** **.** Data is based on methylome results. CG, CHG and CHH methylations are shown on top (positive values) and bottom strand (negative values) across the genes indicated. The corresponding gene models are shown according to TAIR v10. **Figure S16: qRT-PCR expression analysis of At4g00450 and At5g35210.** Error bars are SEM from three technical replicates. **Figure S17:** **qRT-PCR expression analysis of** ***IBM1*** **, expressed as a percentage of mRNA expression in** ***sg1*** **relative to WT.** A gene model representation shows the two mRNA regions assayed, either upstream or downstream of the heterochromatin region located in the large *IBM1* intron. Error bars are SEM from three technical replicates. **Figure S18: Complementation of** ***sg1-1*** **by IBM1.** *ibm1-1* and *sg1-1* were transformed by a construct carrying a functional *IBM1* cDNA [17]. T2 plants were selected on kanamycin, transferred to the greenhouse and pictured after three weeks of growth. **Figure S19: Examples of genes differentially methylated in** ***sg1-1*** **and** ***ibm1*** **.** Graphic representation is based on the publicly available data for *ibm1-6* and the corresponding Col-0 methylome [16]. The genome annotation is based on TAIR v10. The red box indicates the genes differentially methylated. **Figure S20: Examples of genes CHG-methylated in** ***ibm1*** **but not** ***sg1-1*** **.** Graphic representation is based on the publicly available data for *ibm1-6* and the corresponding Col-0 methylome [16]. The genome annotation is based on TAIR v10. The red box indicates the genes differentially methylated.
(PDF)

File S2 **Contains: Table S1. Sequencing statistics. Table S2. Bisulfite sequencing statistics.** Data were obtained by the bismark methylation extractor software. **Table S3. List of genes differentially CHG enriched by at least a factor 2 in** ***sg1-1*** **compared to Col-0.** The ratios between the number of reads containing methylated cytosines and the total number of reads per gene is indicated. The data correspond to the mean from two different biological replicates for both Col-0 and *sg1-1*. Hyper- and hypomethylated genes are listed. Genes selected to analyze both CHG methylation and H3K9 enrichment within genes bodies (Figures 3 and 5) are indicated in red. **Table S4. Members of the RdDM pathway with a CHG-methylation status affected in** ***sg1-1.*** **Table S5. List of oligos used in this study.**
(XLSX)
