# Supplementary material for: SHOOT GROWTH1 Maintains Arabidopsis Epigenomes by Regulating IBM1
Source: PLoS One. 2014 Jan 3;9(1):e84687. doi: 10.1371/journal.pone.0084687 (PMC3880313; doi:10.1371/journal.pone.0084687)

Figure S1

A

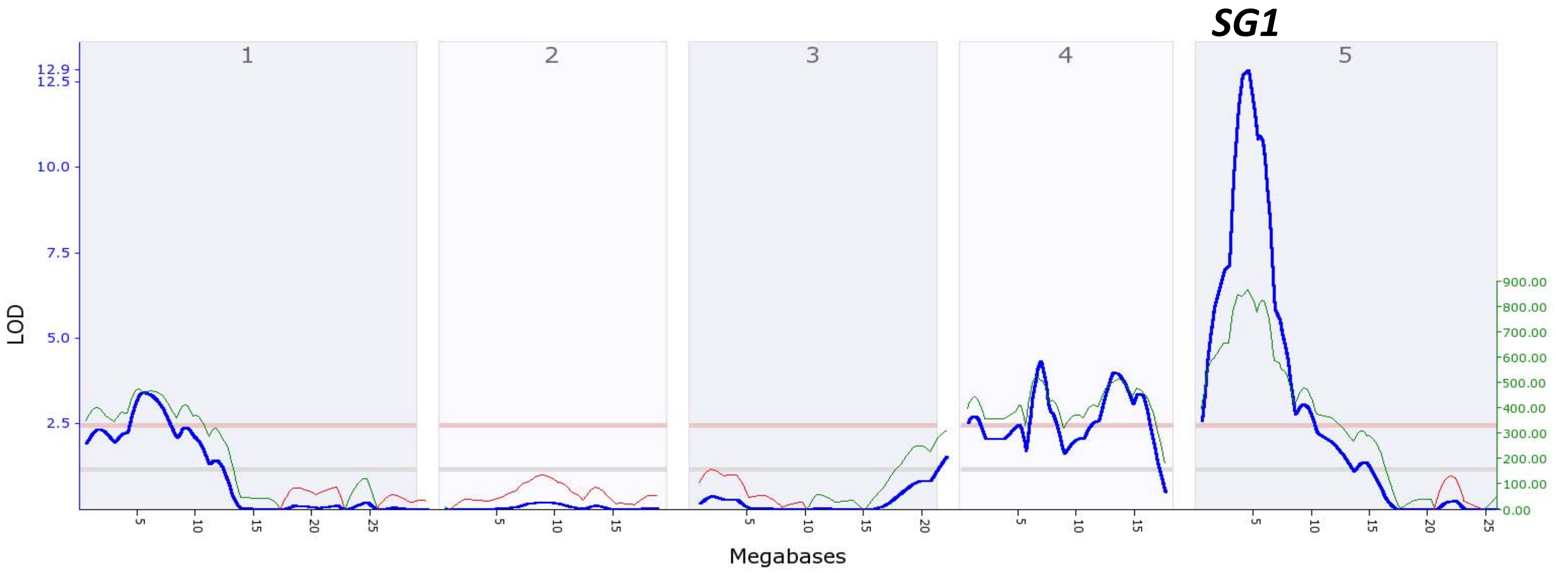
SG1
1
2
3
4
5
LOD
12.9
12.5
10.0
7.5
5.0
2.5
900.00
800.00
700.00
600.00
500.00
400.00
300.00
200.00
100.00
0.00
Megabases

B

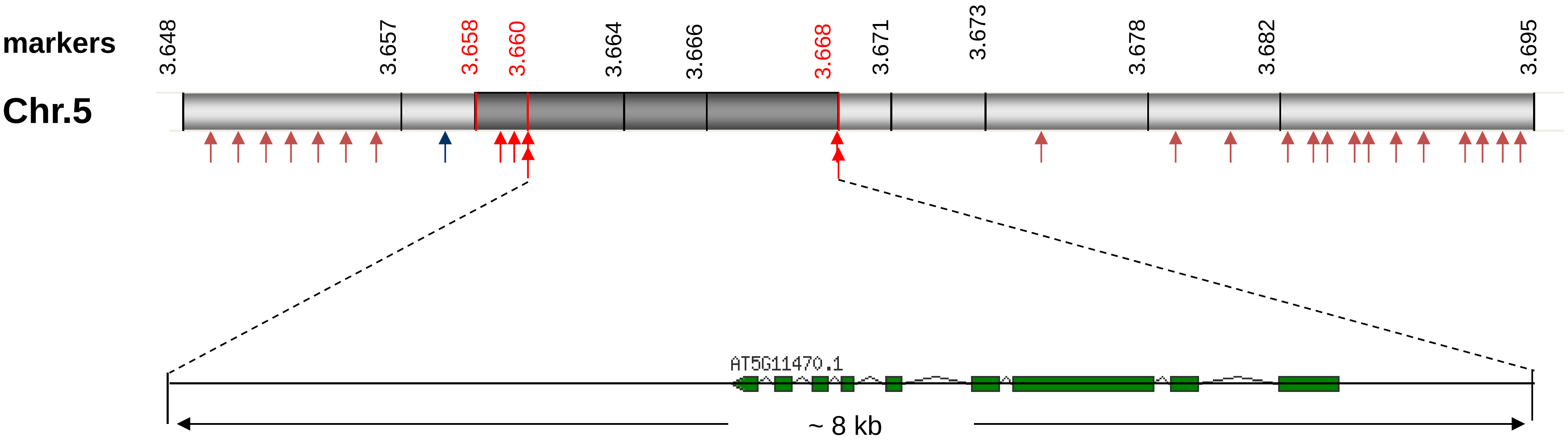
markers
Chr.5
3.648
3.657
3.658
3.660
3.664
3.666
3.668
3.671
3.673
3.678
3.682
3.695
AT5G11470.1
~ 8 kb

Figure S2

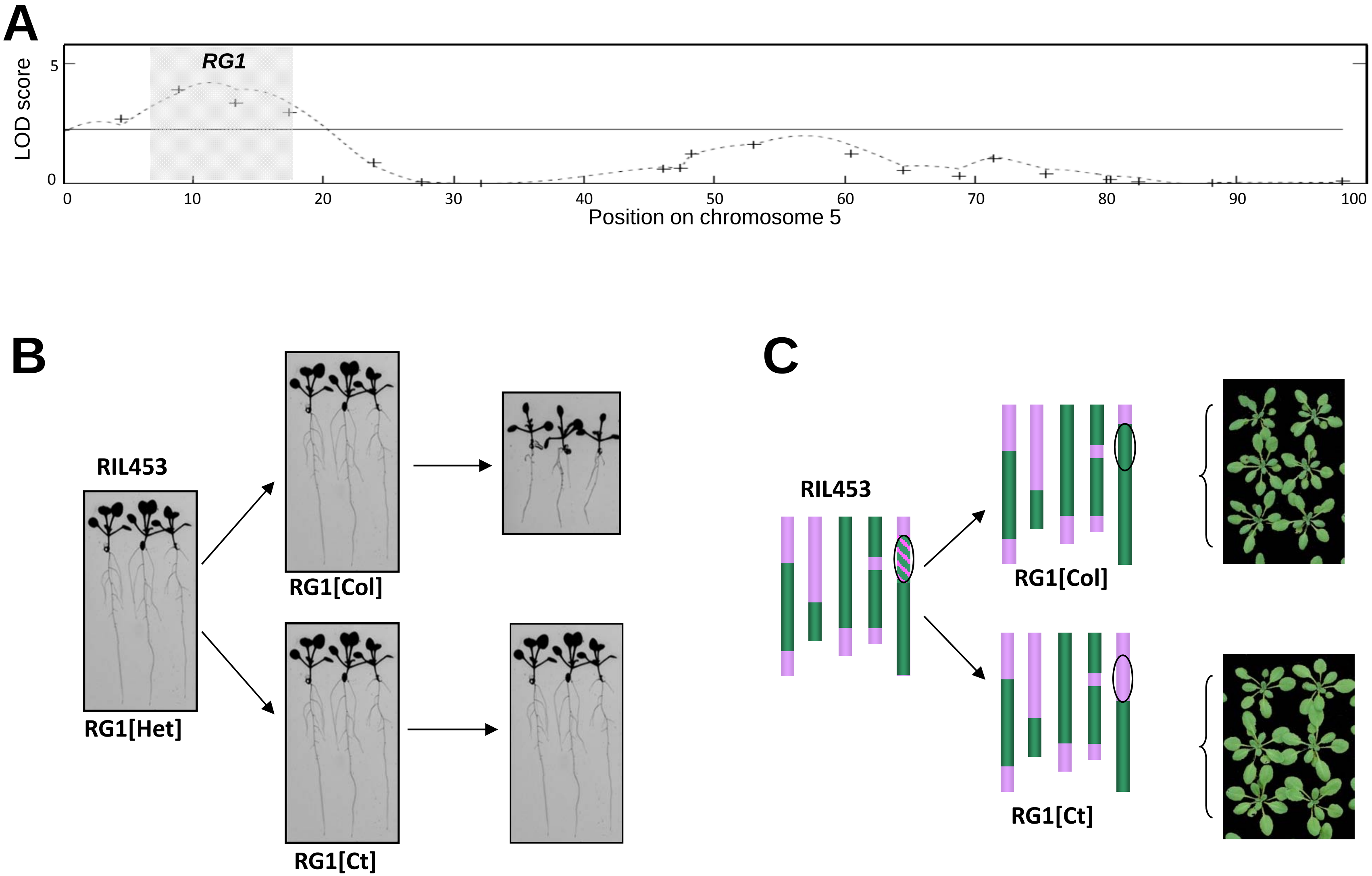
A
RG1
LOD score
5
0
0
10
20
30
40
50
60
70
80
90
100
Position on chromosome 5
B
RIL453
RG1[Het]
RG1[Col]
RG1[Ct]
C
RIL453
RG1[Col]
RG1[Ct]

Figure S3

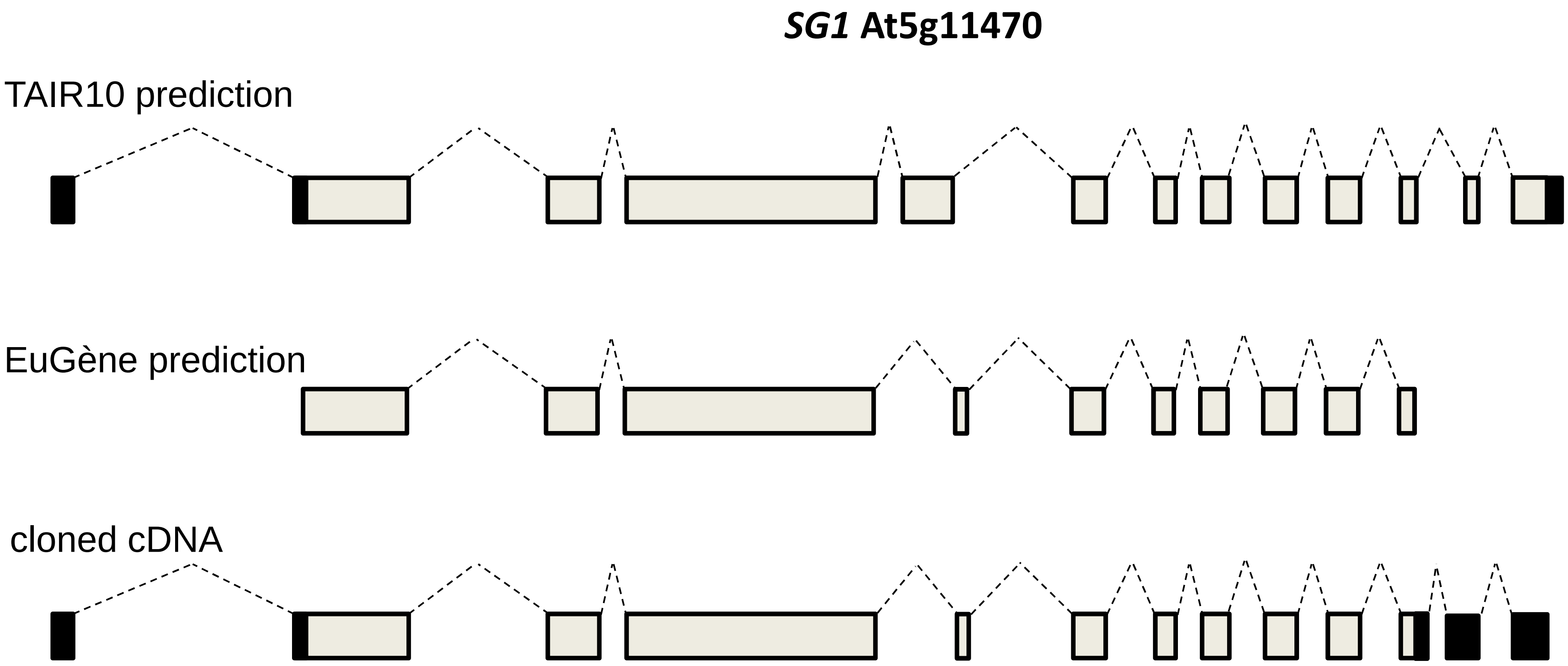
SG1 At5g11470
TAIR10 prediction
EuGène prediction
cloned cDNA

Figure S4

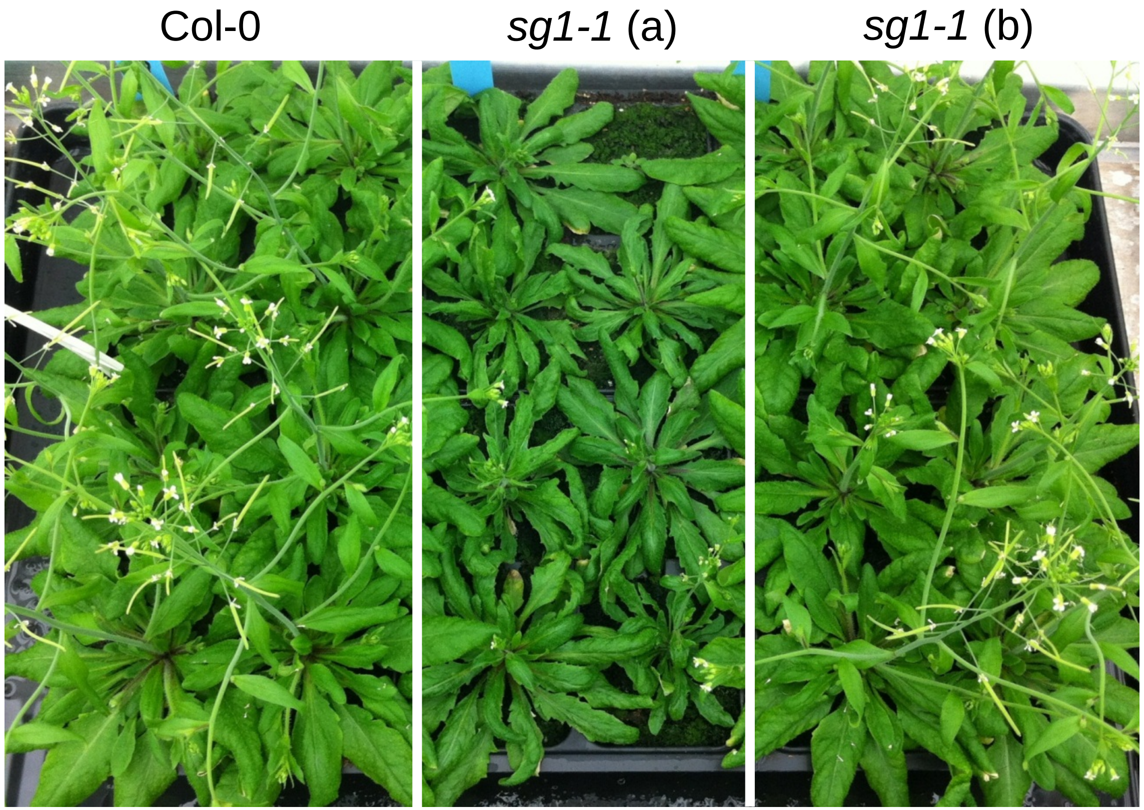

Figure S5

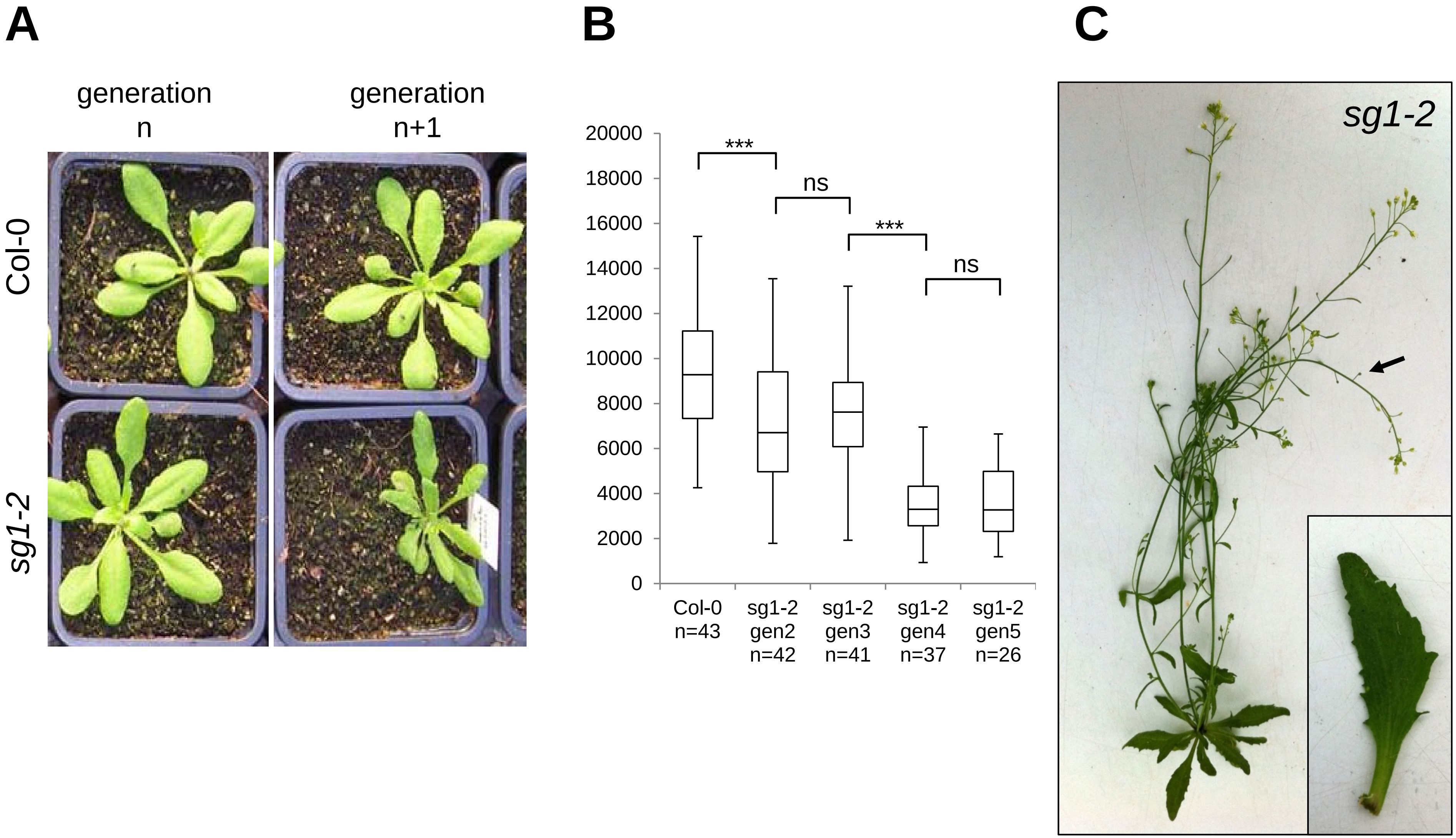
A
generation n
generation n+1
Col-0
sg1-2
B
20000
18000
16000
14000
12000
10000
8000
6000
4000
2000
0
***
ns
***
ns
Col-0 n=43
sg1-2 gen2 n=42
sg1-2 gen3 n=41
sg1-2 gen4 n=37
sg1-2 gen5 n=26
C
sg1-2

Figure S5 (continued)

D

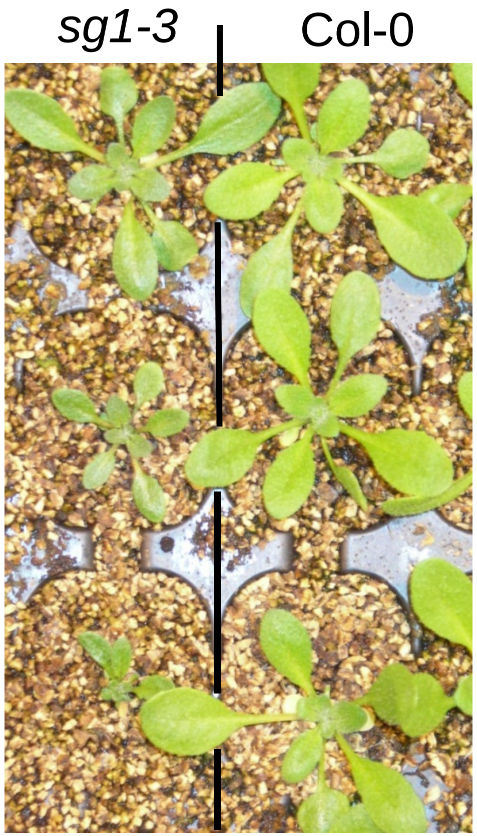

Figure S6

CMT1 (At1g80740), CMT3(At1g69770)

CMT2 (At4g19020)

DMT2 (At4g14140), DNMT1 (At4g13610),
MET1 (At5g49160), MET2 (At4g08990)

AtORC1a (At4g14700)

At ORC1b (At4g12620)

UNCHARACTERIZED PROTEIN: At4g11560

UNCHARACTERIZED PROTEINS: At3g48050, At3g48060

ES43 like proteins: At4g39100, At4g22140

SG1 (At5g11470)

UNCHARACTERIZED PROTEINS: At1g68580, At5g55600

UNCHARACTERIZED PROTEIN: At4g23120, At2g25120,
At3g43990, At2g04260

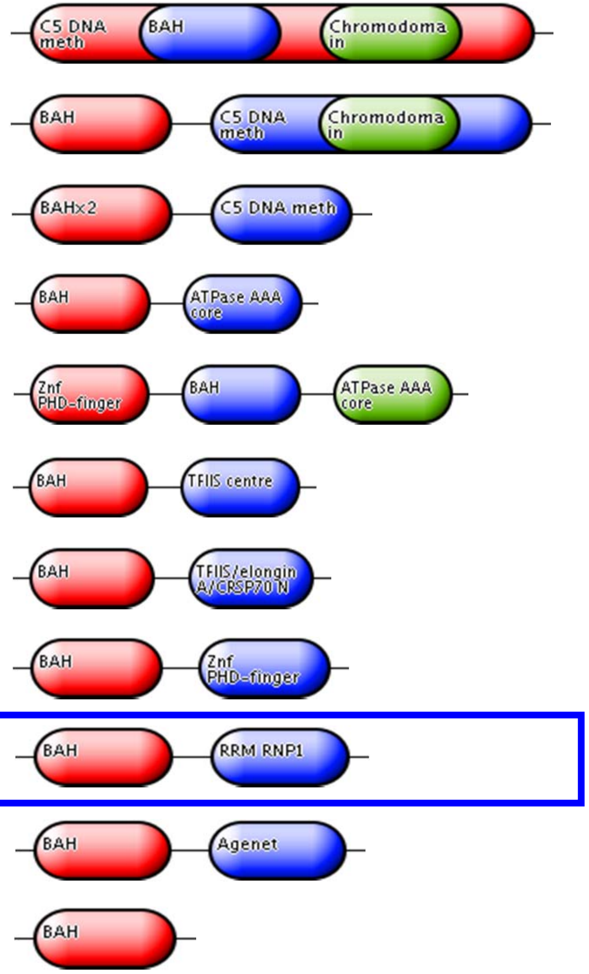

Figure S7

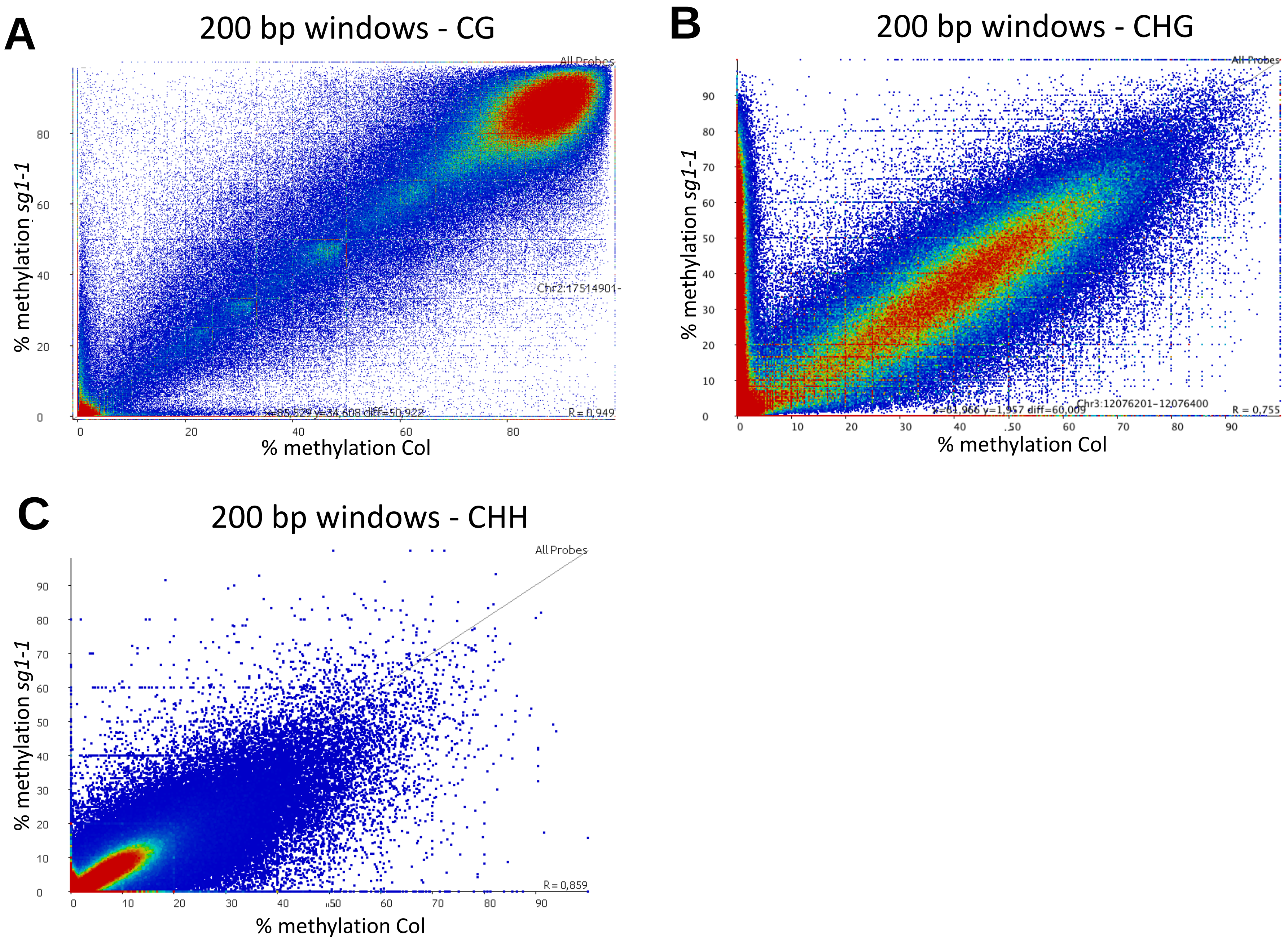

A
200 bp windows - CG
All Probes
Chr2:17514901-
R = 0,949
% methylation sg1-1
% methylation Col
0
20
40
60
80
B
200 bp windows - CHG
All Probes
Chr3:12076201-12076400
x=61,966 y=1,957 diff=60,009
R = 0,755
% methylation sg1-1
% methylation Col
0
10
20
30
40
50
60
70
80
90
C
200 bp windows - CHH
All Probes
R = 0,859
% methylation sg1-1
% methylation Col
0
10
20
30
40
50
60
70
80
90

Figure S8

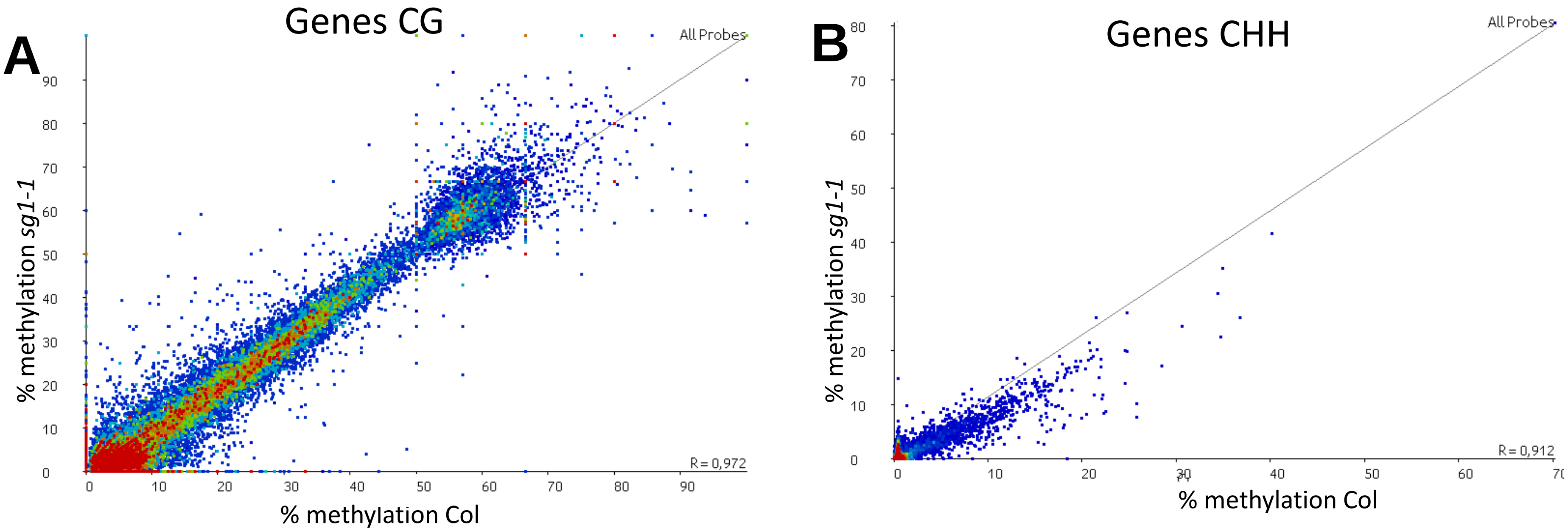
A
Genes CG
All Probes
% methylation sg1-1
% methylation Col
R = 0,972
0
10
20
30
40
50
60
70
80
90
B
Genes CHH
All Probes
% methylation sg1-1
% methylation Col
R = 0,912

Figure S9

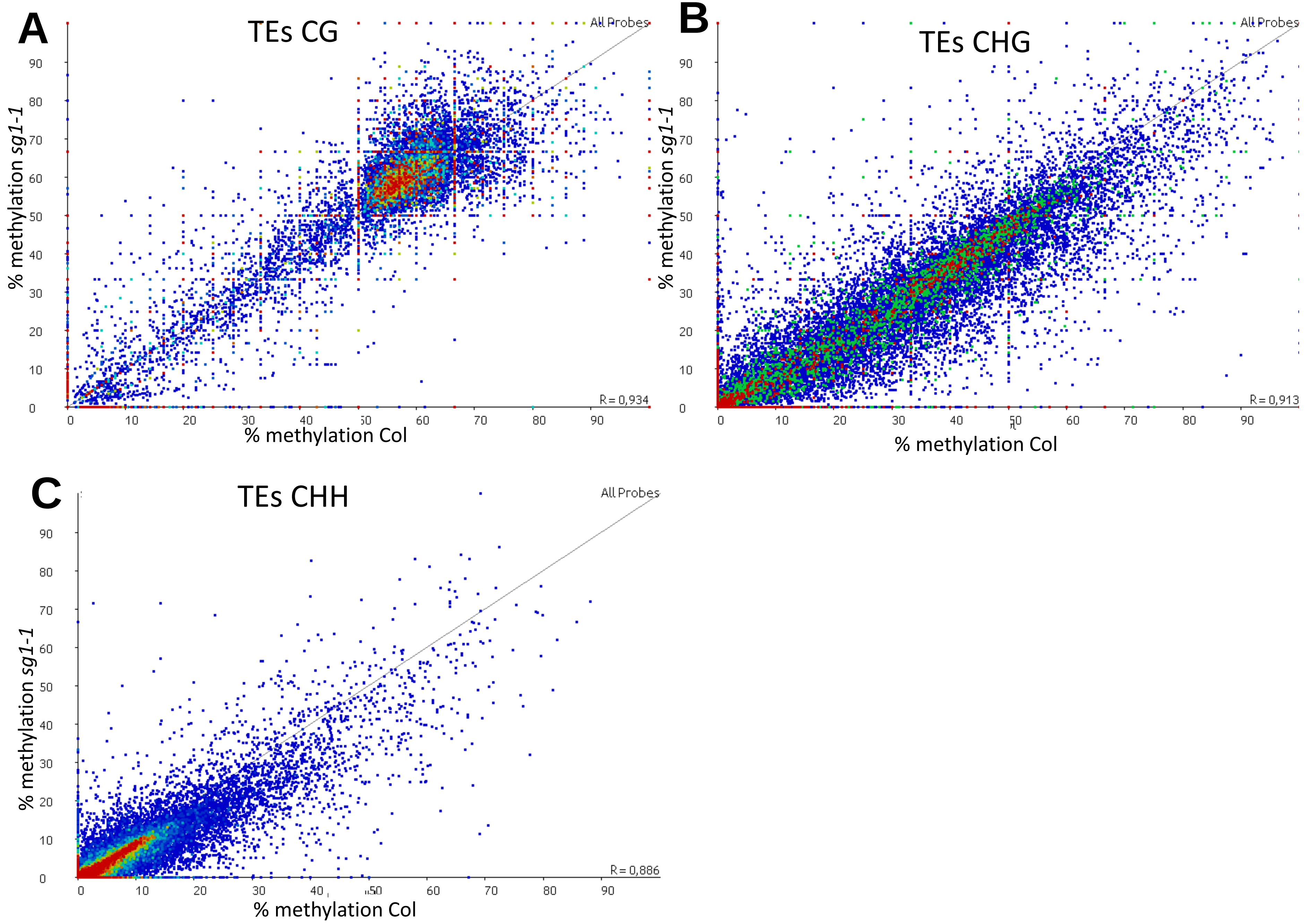
A
TEs CG
All Probes
R = 0,934
% methylation sg1-1
% methylation Col
B
TEs CHG
All Probes
R = 0,913
% methylation sg1-1
% methylation Col
C
TEs CHH
All Probes
R = 0,886
% methylation sg1-1
% methylation Col

Figure S10

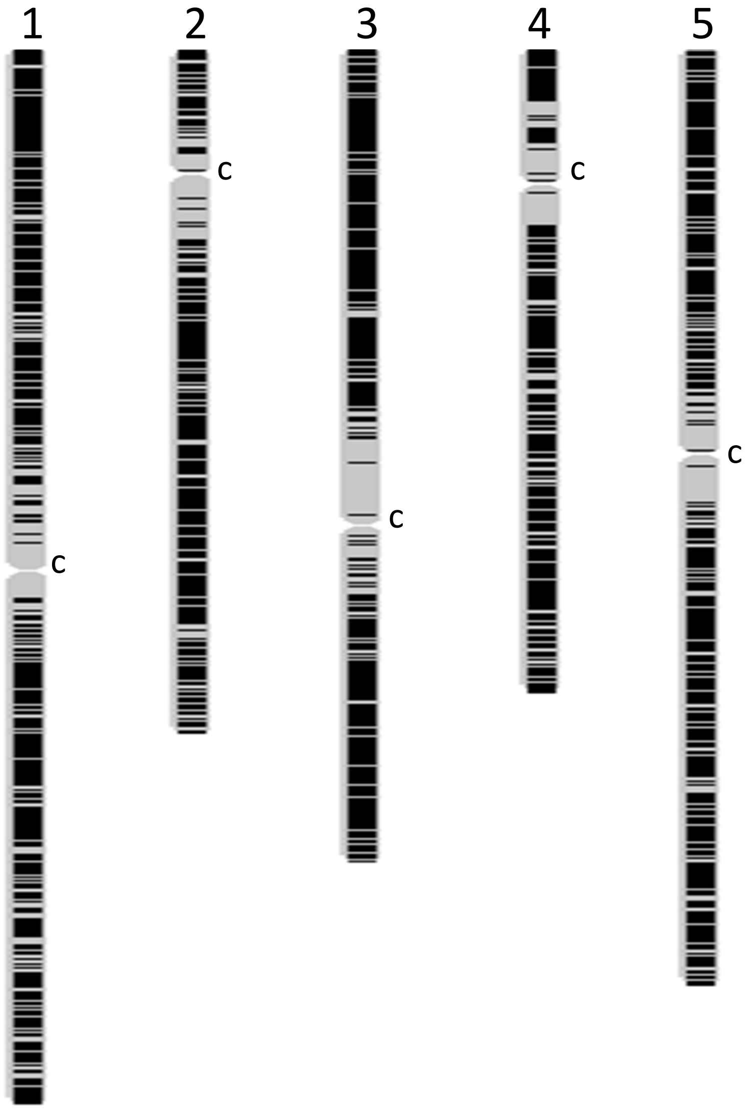

Figure S11

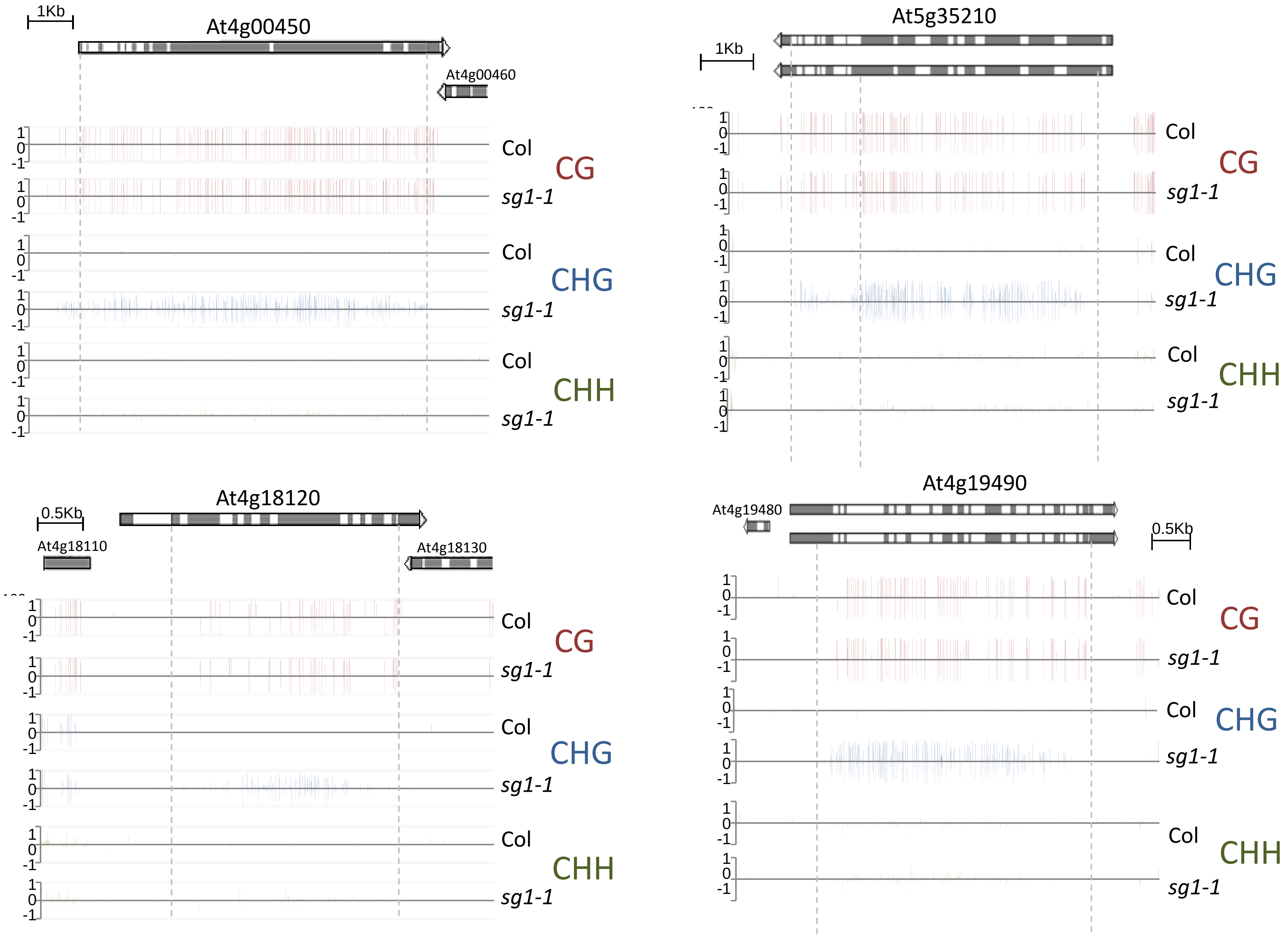
1Kb
At4g00450
At4g00460
Col
sg1-1
CG
CHG
CHH
At5g35210
0.5Kb
At4g18120
At4g18110
At4g18130
At4g19490
At4g19480
1
0
-1

Figure S11 (continued)

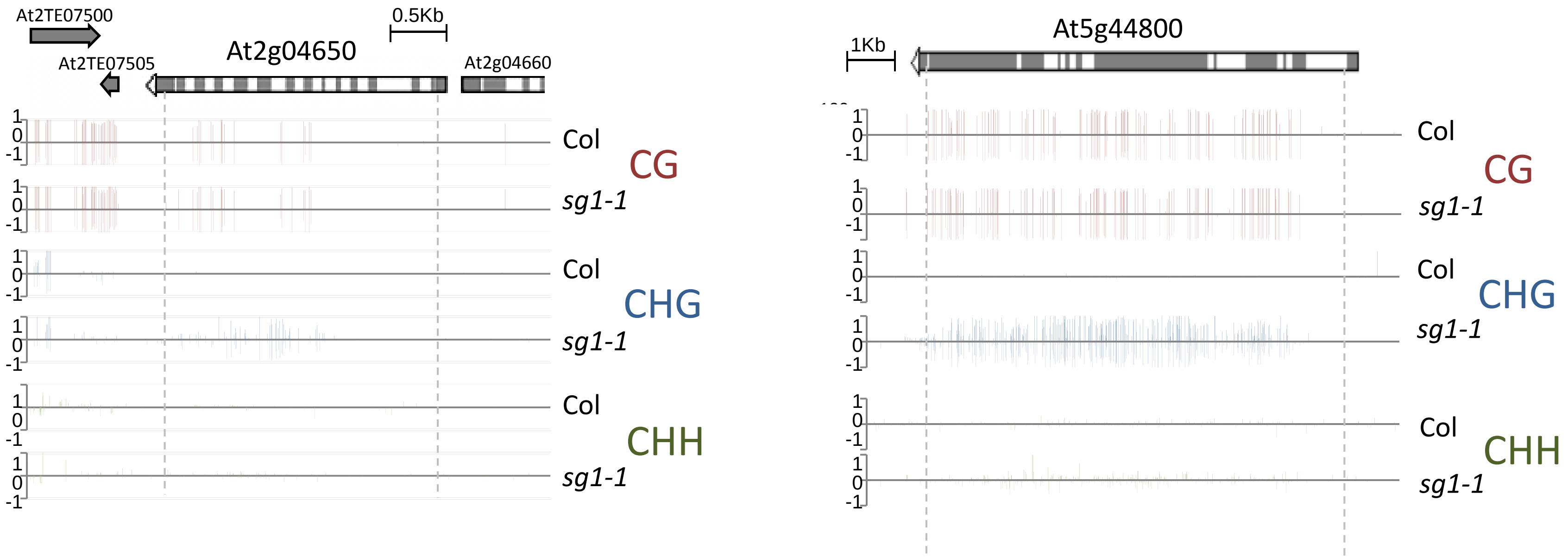

Figure S12

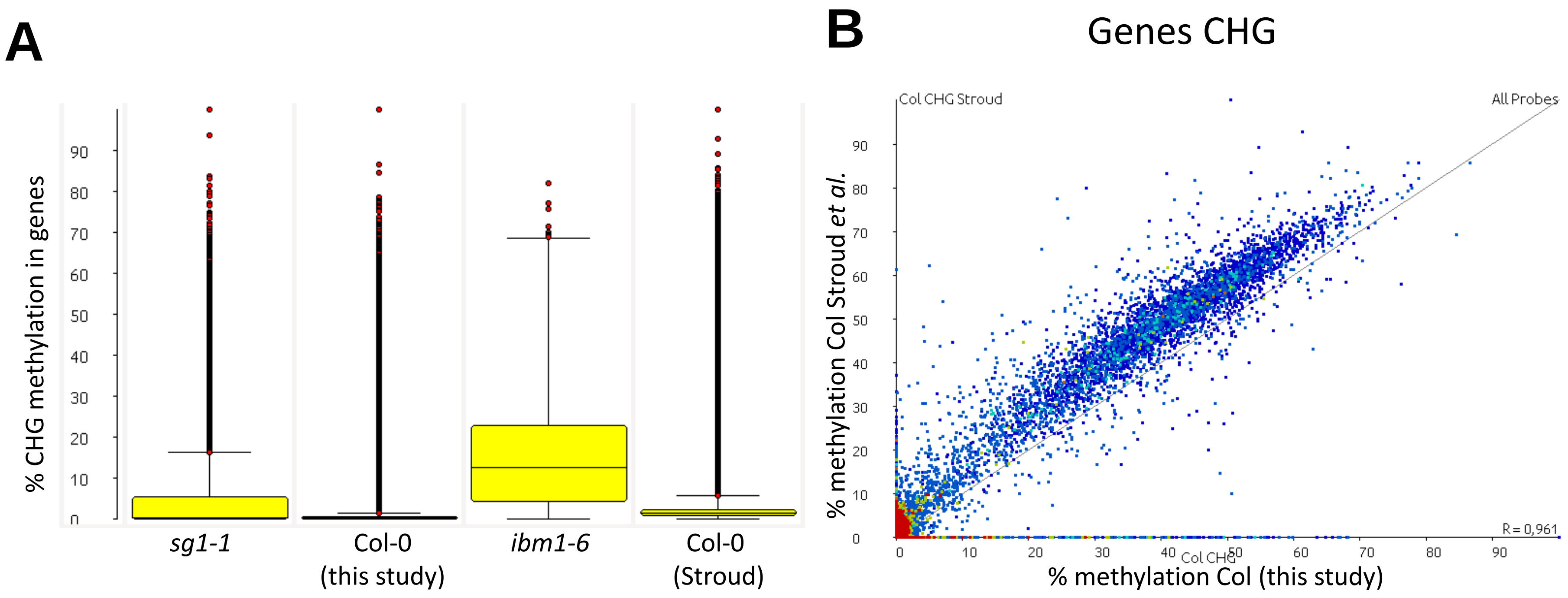
A
% CHG methylation in genes
90
80
70
60
50
40
30
20
10
0
sg1-1
Col-0 (this study)
ibm1-6
Col-0 (Stroud)
B
Genes CHG
Col CHG Stroud
All Probes
% methylation Col Stroud et al.
R = 0,961
Col CHG
% methylation Col (this study)

Figure S13

A

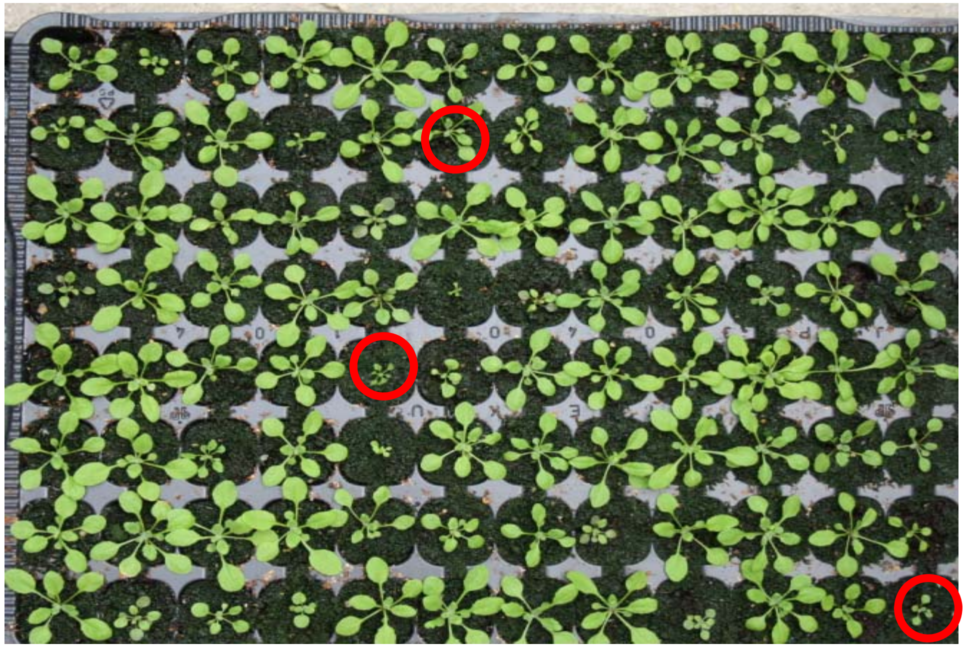

B

Col-0 ***met1-1*** ***sg1-2*** ***met1-1/sg1-2***

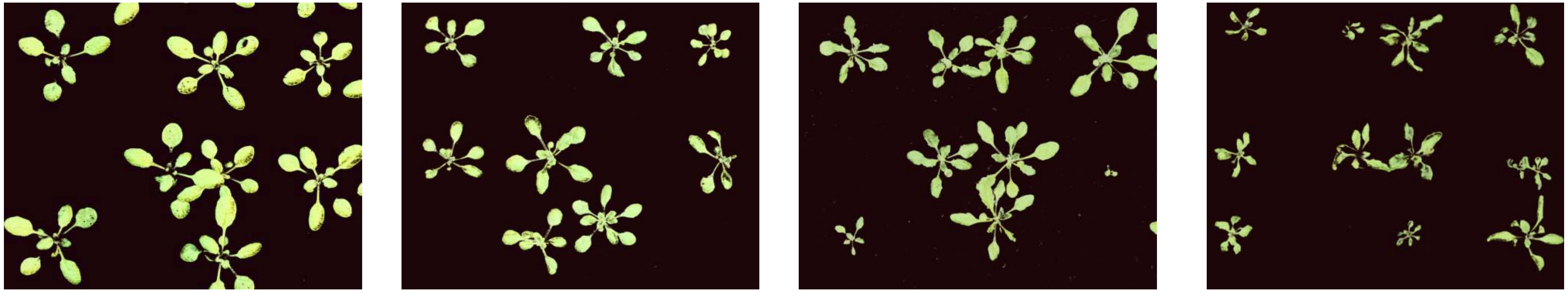

Figure S14

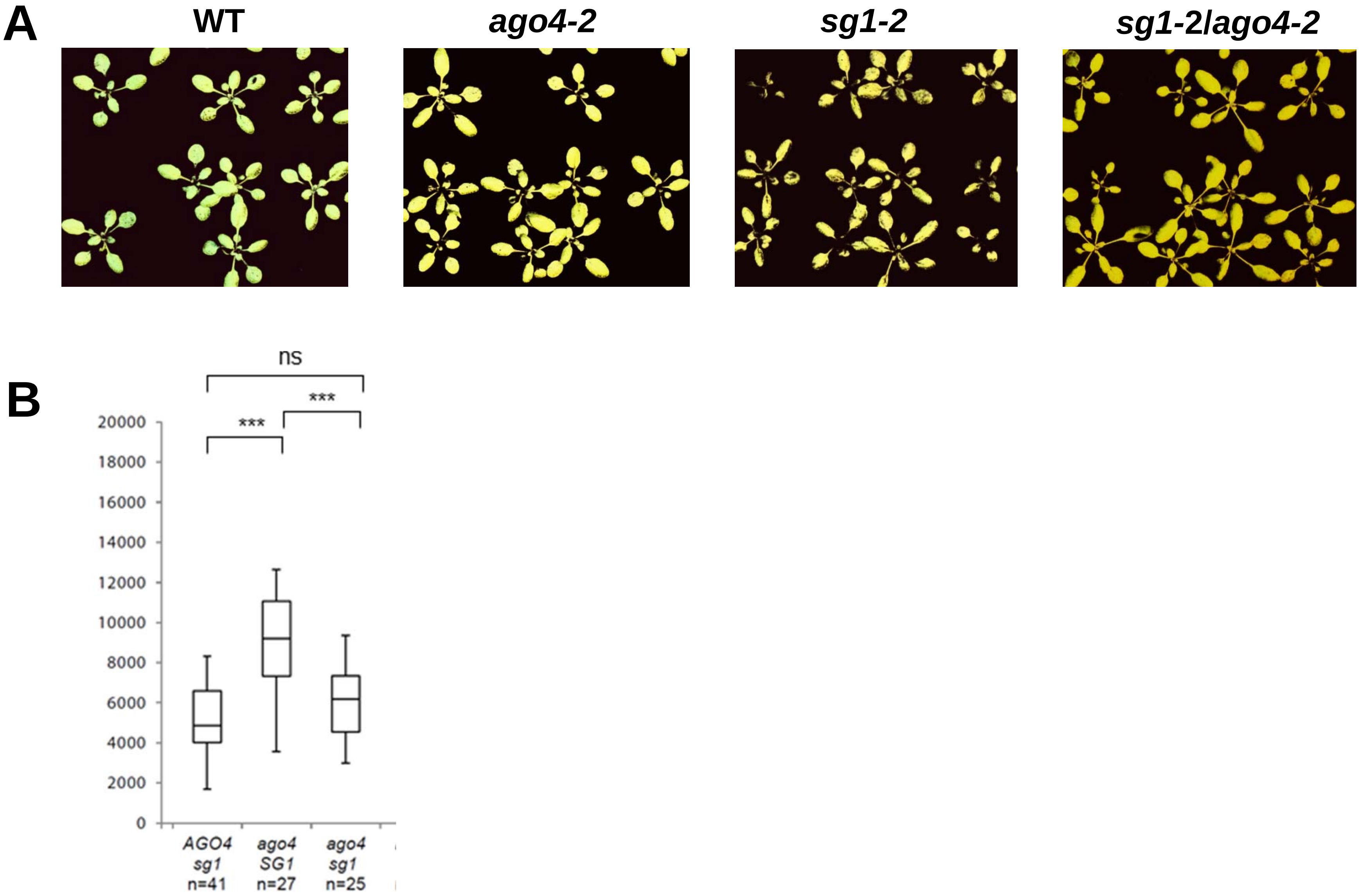

A
WT
ago4-2
sg1-2
sg1-2/ago4-2
B
ns
***
***
20000
18000
16000
14000
12000
10000
8000
6000
4000
2000
0
AGO4 sg1 n=41
ago4 SG1 n=27
ago4 sg1 n=25

Figure S15

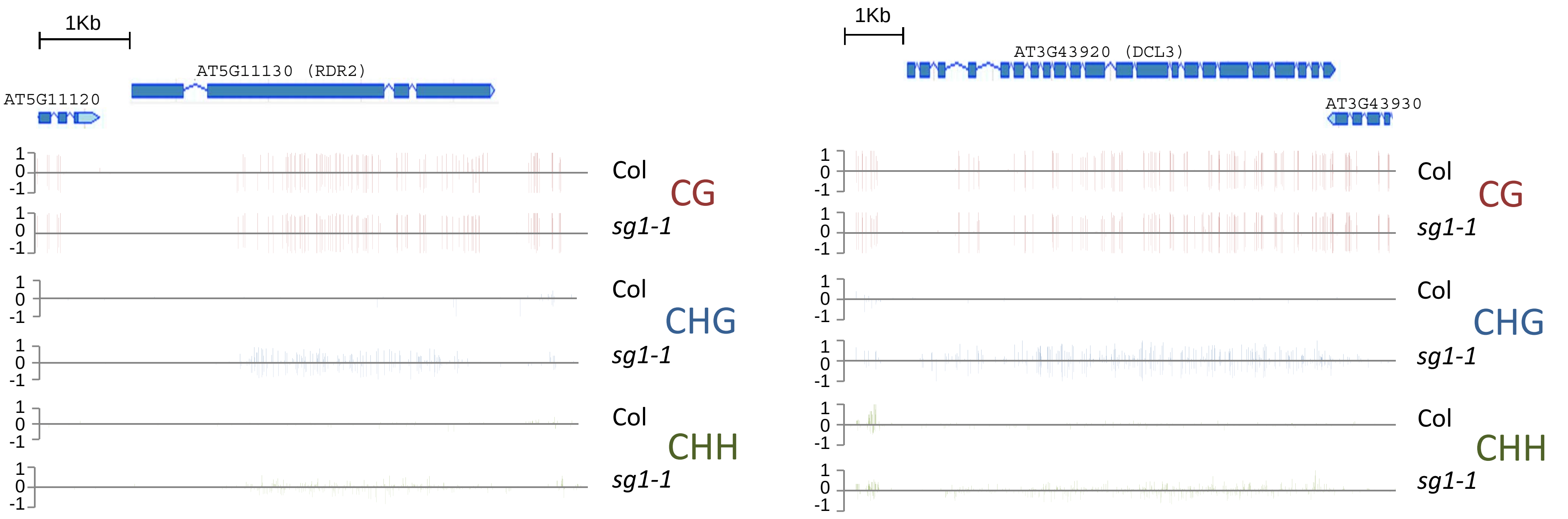

1Kb
AT5G11130 (RDR2)
AT5G11120
1Kb
AT3G43920 (DCL3)
AT3G43930
Col
CG
sg1-1
Col
CHG
sg1-1
Col
CHH
sg1-1

Figure S16

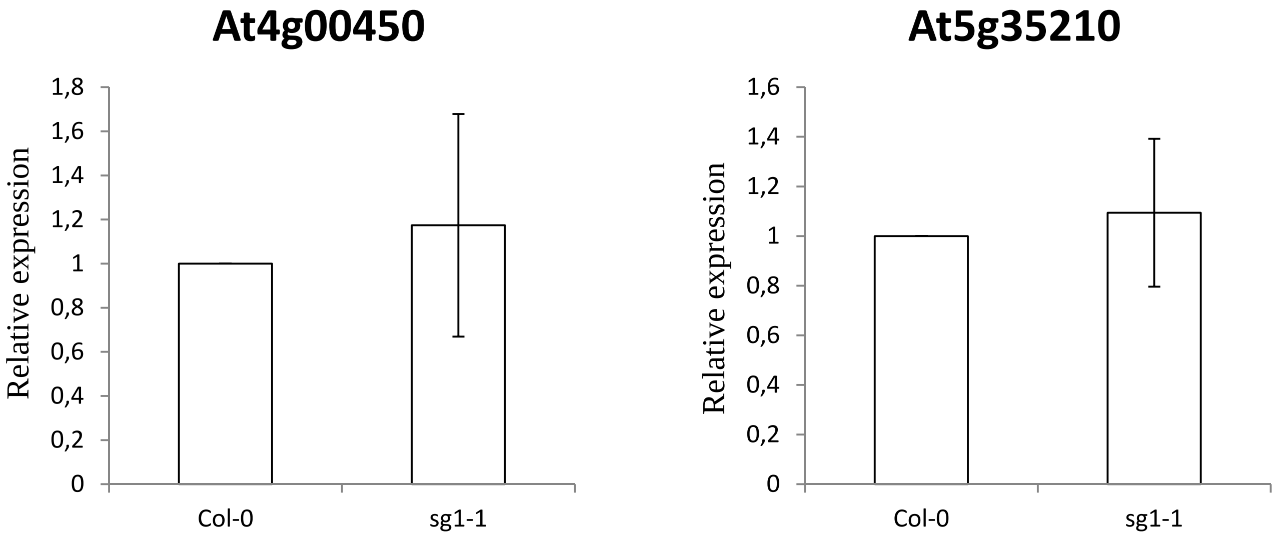
At4g00450
Relative expression
1,8
1,6
1,4
1,2
1
0,8
0,6
0,4
0,2
0
Col-0
sg1-1
At5g35210
Relative expression
1,6
1,4
1,2
1
0,8
0,6
0,4
0,2
0
Col-0
sg1-1

Figure S17

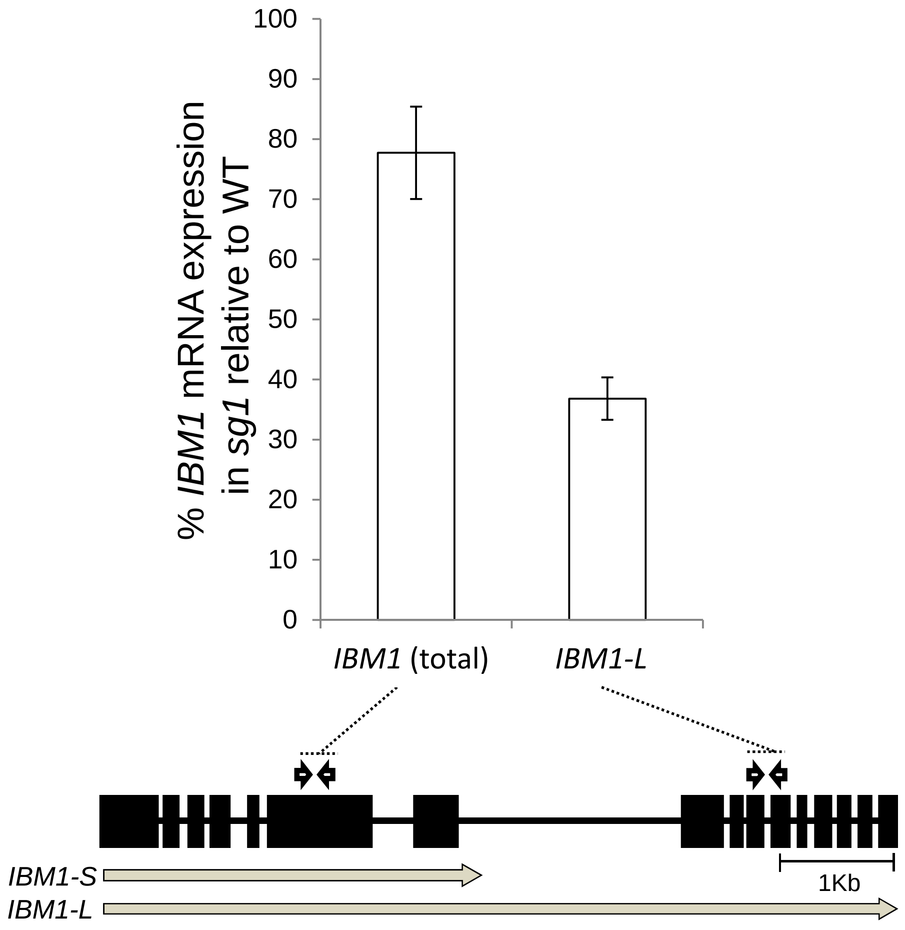

100
90
80
70
60
50
40
30
20
10
0
% IBM1 mRNA expression in sg1 relative to WT
IBM1 (total)
IBM1-L
IBM1-S
IBM1-L
1Kb

Figure S18

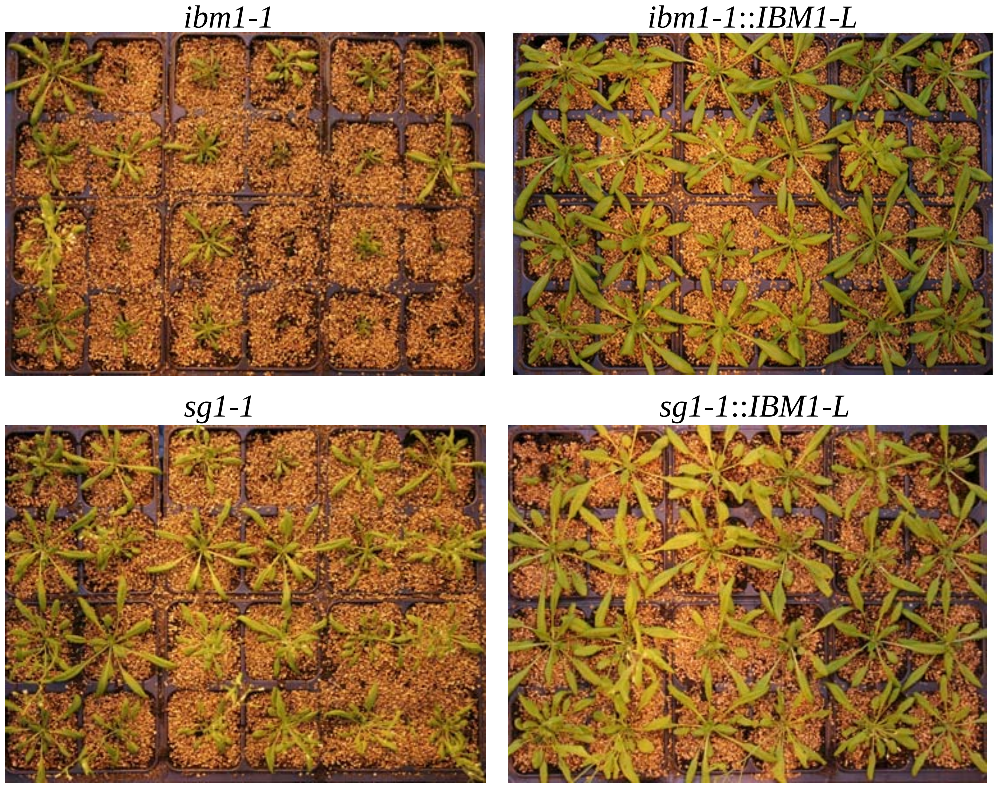

Figure S19

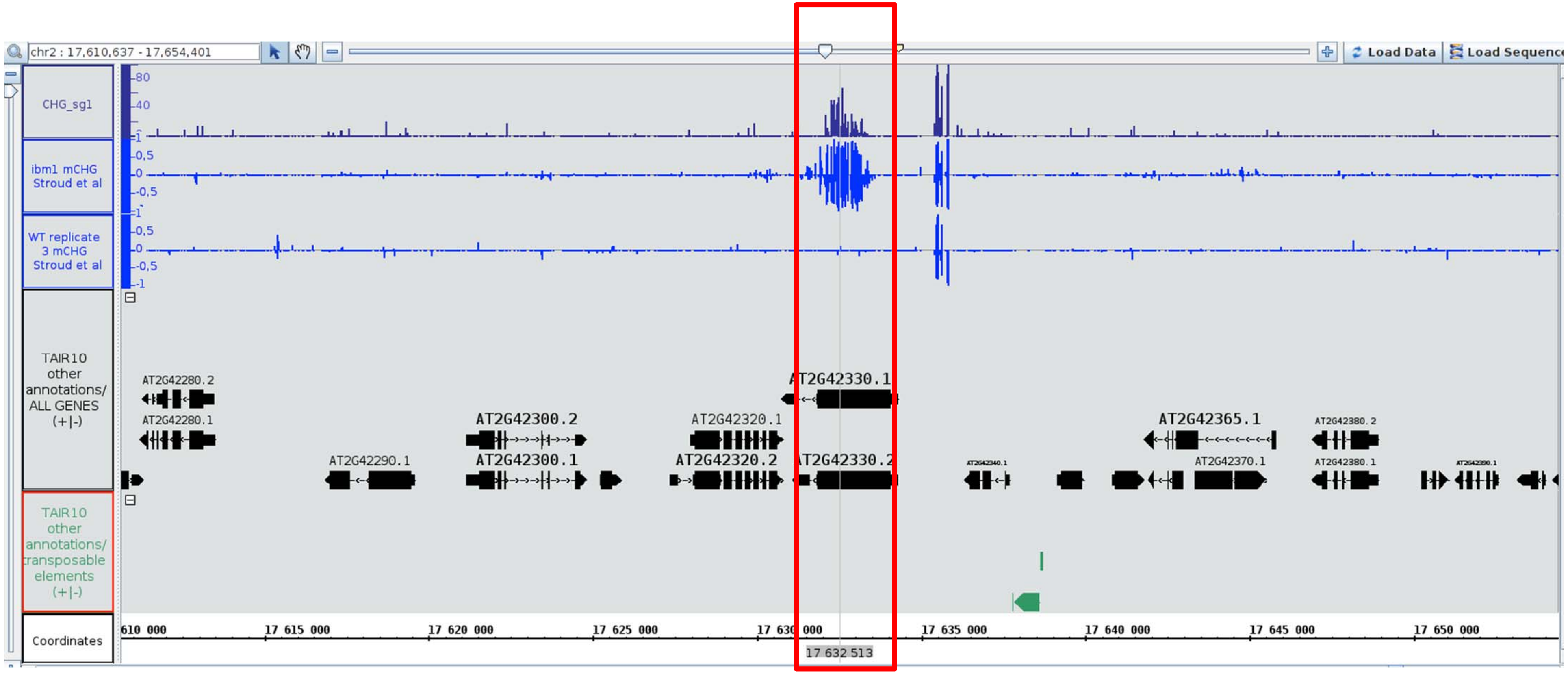
chr2 : 17,610,637 - 17,654,401
Load Data
Load Sequence
CHG_sg1
ibm1 mCHG Stroud et al
WT replicate 3 mCHG Stroud et al
TAIR10 other annotations/ ALL GENES (+|-)
TAIR10 other annotations/ transposable elements (+|-)
Coordinates
AT2G42280.2
AT2G42280.1
AT2G42290.1
AT2G42300.2
AT2G42300.1
AT2G42320.1
AT2G42320.2
AT2G42330.1
AT2G42330.2
AT2G42365.1
AT2G42370.1
AT2G42380.2
AT2G42380.1
610 000
17 615 000
17 620 000
17 625 000
17 635 000
17 640 000
17 645 000
17 650 000
17 632 513

Figure S20

A

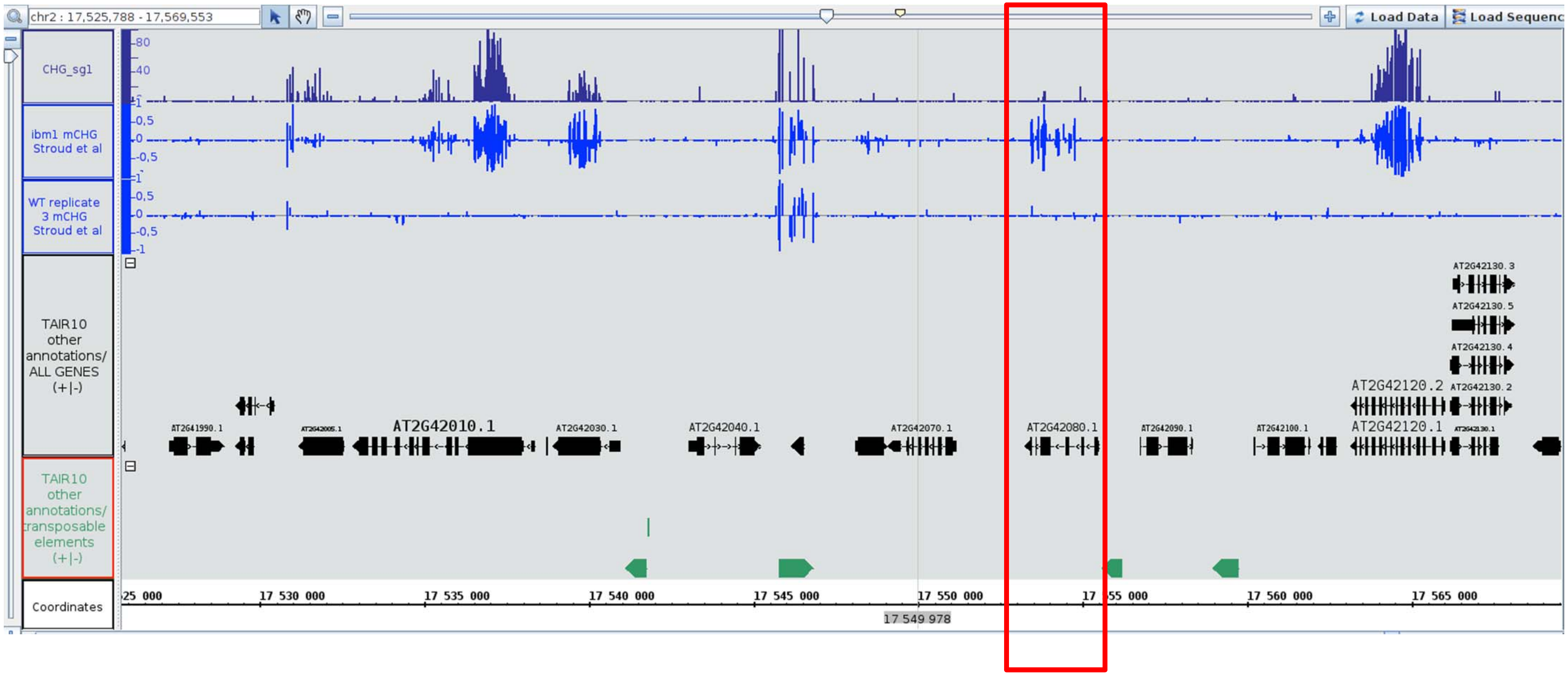

chr2 : 17,525,788 - 17,569,553
Load Data
Load Sequenc
CHG_sgl
ibm1 mCHG Stroud et al
WT replicate 3 mCHG Stroud et al
TAIR10 other annotations/ ALL GENES (+|-)
TAIR10 other annotations/ transposable elements (+|-)
Coordinates
AT2G41990.1
AT2G42010.1
AT2G42030.1
AT2G42040.1
AT2G42070.1
AT2G42080.1
AT2G42090.1
AT2G42100.1
AT2G42120.2
AT2G42120.1
AT2G42130.3
AT2G42130.5
AT2G42130.4
AT2G42130.2
17 530 000
17 535 000
17 540 000
17 545 000
17 550 000
17 560 000
17 565 000
17 549 978

Figure S20 (continued)

B

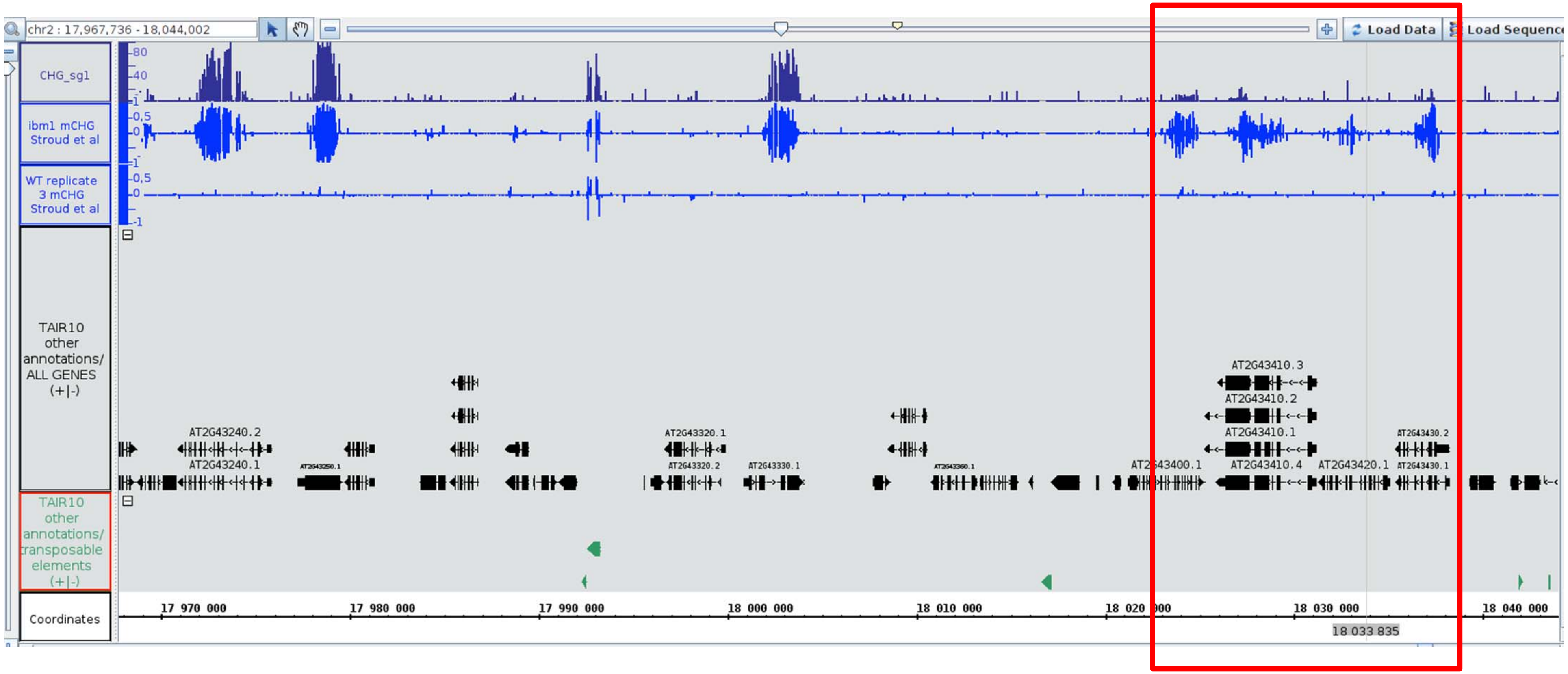

Supplement: File S1 — Contains: Figure S1: SG1 fine mapping. (A) QTL mapping for shoot size in the Bur-0 x Col-0 RIL set identifies a major QTL on chromosome 5 [12]. (B) Fine-mapping of the major-effect locus to 8 kb around At5g11470 on chromosome 5. Figure S2: RG1 fine mapping in the Ct-1 x Col-0 maps to SG1. QTL mapping for root growth (total root length) in the Ct-1 x Col-0 RIL set (A) reveals RG1, a QTL confirmed in vitro 10 days after germination (days after germination) (B) and in the greenhouse 20 days after germination (C). Fine-mapping and sequencing revealed that sg1-1 mutation is responsible for this QTL. Figure S3: SG1 gene models. SG1 gene models according to TAIR v10, EuGène and our cDNA sequencing data is shown with UTRs (black boxes), exons (clear boxes) and introns (dashed lines). Left, 5′ end; right, 3′ end. Figure S4: sg1 phenotype at the flowering stage. Picture of a tray of 40 days-old WT Col-0 plants (left) and sg1-1 plants (middle, right) derived from a parent exhibiting (a) or not (b) a phenotype. The flowering delay can be seen for sg1-1 (a). Figure S5: sg1-2 and sg1-3 mutants. (A) Phenotype of plants homozygous for sg1-2 T-DNA (bottom) and WT Col-0 (top) in two successive generations after fixation at the homozygous state. (B) Analysis of the projected rosette area (pixels) of 15 days-old seedlings grown in vitro. sg1-2(gen2): second homozygous generation. Each following generation (gen3, gen4 and gen5) descends from the previous one. ***p<0.001, ns: not significant. (C) Picture of a mature sg1-2 plant. The arrow points one sterile bud that failed to develop into a silique. The frame shows an enlargement of a rosette leaf showing the over-serrated shape. (D) Segregating plants (left) originating from a unique sg1-3 homozygous parent illustrate the stochasticity of the sg1 phenotype compared to segregating plants from a unique WT Col-0 parent (right). Figure S6: Proteins containing Bromo adjacent homology (BAH) domain (IPR001025) in Arabidopsis. The different dom [file pone.0084687.s001.pdf]
